# Application of a cocktail approach to screen cytochrome P450 BM3 libraries for metabolic activity and diversity

**DOI:** 10.1007/s00216-015-9241-x

**Published:** 2016-01-11

**Authors:** Jelle Reinen, Geert Postma, Cornelis Tump, Tom Bloemberg, Jasper Engel, Nico P. E. Vermeulen, Jan N. M. Commandeur, Maarten Honing

**Affiliations:** Division of Molecular Toxicology, Amsterdam Institute for Molecules Medicines and Systems (AIMMS), Faculty of Sciences, VU University Amsterdam, De Boelelaan 1083, 1081 HV Amsterdam, The Netherlands; Institute for Molecules and Materials, Analytical Chemistry, Radboud University Nijmegen, P.O. Box 9010, 6500 GL Nijmegen, The Netherlands; QPS Netherlands B.V., Petrus Campersingel 123, 9713 AG Groningen, The Netherlands; Division of BioAnalytical Chemistry, Amsterdam Institute for Molecules Medicines and Systems (AIMMS), Faculty of Sciences, VU University Amsterdam, De Boelelaan 1083, 1081 HV Amsterdam, The Netherlands; DSM Resolve, Urmonderbaan 22, 6160 MD Geleen, The Netherlands

**Keywords:** Cytochrome P450 BM3, Cocktail screening approach, Chemometrics, Biocatalysis, LC-MS(/MS)

## Abstract

In the present study, the validity of using a cocktail screening method in combination with a chemometrical data mining approach to evaluate metabolic activity and diversity of drug-metabolizing bacterial Cytochrome P450 (CYP) BM3 mutants was investigated. In addition, the concept of utilizing an in-house-developed library of CYP BM3 mutants as a unique biocatalytic synthetic tool to support medicinal chemistry was evaluated. Metabolic efficiency of the mutant library towards a selection of CYP model substrates, being amitriptyline (AMI), buspirone (BUS), coumarine (COU), dextromethorphan (DEX), diclofenac (DIC) and norethisterone (NET), was investigated. First, metabolic activity of a selection of CYP BM3 mutants was screened against AMI and BUS. Subsequently, for a single CYP BM3 mutant, the effect of co-administration of multiple drugs on the metabolic activity and diversity towards AMI and BUS was investigated. Finally, a cocktail of AMI, BUS, COU, DEX, DIC and NET was screened against the whole in-house CYP BM3 library. Different validated quantitative and qualitative (U)HPLC-MS/MS-based analytical methods were applied to screen for substrate depletion and targeted product formation, followed by a more in-depth screen for metabolic diversity. A chemometrical approach was used to mine all data to search for unique metabolic properties of the mutants and allow classification of the mutants. The latter would open the possibility of obtaining a more in-depth mechanistic understanding of the metabolites. The presented method is the first MS-based method to screen CYP BM3 mutant libraries for diversity in combination with a chemometrical approach to interpret results and visualize differences between the tested mutants.

**Electronic supplementary material:**

The online version of this article (doi:10.1007/s00216-015-9241-x) contains supplementary material, which is available to authorized users.

**Graphical abstract:**
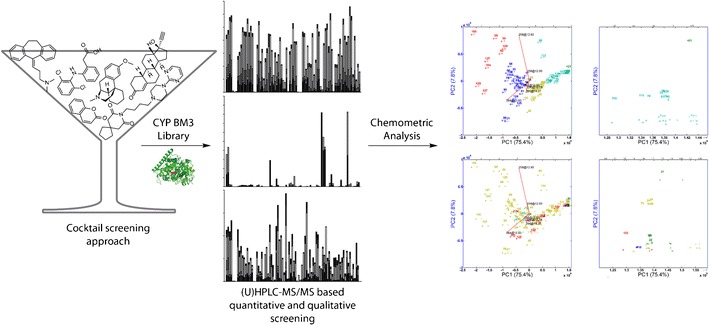
General worklfow in screening mutant enzyme libraries for catalytic efficiency and diversity

## Introduction

Cytochrome P450 monooxygenases (CYPs) play an essential role in metabolism of a wide range of xenobiotics and are involved in biosynthesis of prostaglandins and steroid hormones. CYPs catalyze hydroxylation, epoxidation, reduction and other oxidative reactions on substrates that range from alkanes to complex endogenous molecules such as drugs, steroids and fatty acids [1]. The many reactions that CYPs can catalyze make these enzymes highly versatile and very interesting as tools to generate rather diverse and unique medicinal compound libraries not always accessible via classical synthetic routes. Moreover, in many cases these compounds show improved physico-chemical properties which is of crucial importance during development of drug dosage forms. Metabolites produced by CYPs are well known to display important pharmacological activities or may be responsible for the toxicity or other unwanted side effects of xenobiotics [2, 3]. Systems enabling facile biosynthesis of sufficient quantities of metabolites for structural elucidation and pharmacological and toxicological evaluation are therefore highly desirable. In addition, for drug discovery purposes metabolites are very interesting during lead optimization as they may display improved properties, such as target selectivity or even solubility without significant loss of the pharmacological properties compared to the parent drug itself [4].

The ideal biocatalyst possesses high turnover rates, is very stable, can easily be expressed at high levels, and can function under extreme conditions of temperature, pH, buffer system or solvent. Although mammalian CYPs are capable of metabolizing a wide array of substrates, they require additional redox partners, display relatively low activities, give low expression yields and have poor stability which makes them less suitable for biocatalytic purposes [5]. The fatty acid-metabolizing CYP BM3 from *Bacillus megaterium* is a very interesting candidate because it is a natural fusion between a heme domain responsible for substrate oxidation and a diflavin reductase domain responsible for electron transport [6]. CYP BM3 possesses the highest activity ever measured and is a highly stable enzyme [7]. These properties, in combination with availability of crystal structures and large-scale expression and purification protocols, make CYP BM3 a highly suitable candidate for biocatalytic applications. Various protein engineering techniques have already been successfully employed to generate CYP BM3 mutants with increased activities and altered regio- and stereoselectivities [8].

An important step during development of novel CYP BM3 mutants for biocatalytic purposes is selection of mutants that display the desired changes. Therefore, mutant libraries which have been created using different protein engineering techniques need to be screened to select mutants with improved characteristics. In order to improve throughput for screening of CYP BM3 mutant libraries for different substrates, it may be useful to screen the different substrates in a cocktail format. Such an approach is already being used to study inhibitory effects of drugs and new chemical entities (NCE) on human CYP activities using a mixture of CYP-specific substrates in a single human microsomal incubation in combination with tandem mass spectrometry. These cocktail approaches have been shown to be less time-consuming, less labour-intensive, more cost-effective and thereby very powerful tools to study drug-drug interactions due to CYP inhibition [9–13].

The aim of the present study was to investigate if a cocktail approach could be used to screen CYP BM3 libraries for metabolic activity and diversity. Furthermore, it was of interest to know if such an approach could be used in combination with different chemometric tools in order to identify metabolites without prior knowledge of their structure and identity. Firstly, metabolic activity of a selection of well-characterized BM3 mutants from an in-house library was screened against two drugs for which also metabolic profiles were determined. Secondly, for a single BM3 mutant, the effect of co-administration of multiple drugs on the metabolic activity and diversity was investigated. Based on these experiments, a cocktail of drugs was selected against which the whole in-house CYP BM3 mutant library was screened. A chemometrical approach was subsequently used to analyse the data generated by the qualitative HPLC-MS/MS-based screening in order to determine if metabolites without prior knowledge of their structure and identity could be detected. It was demonstrated that a cocktail approach can be utilized to screen CYP BM3 libraries for metabolic activity and diversity and that a chemometrical approach is beneficial in order to visualize and analyse the results generated during the library screen.

## Materials and methods

### Chemicals

5-Hydroxybuspirone and 16β-hydroxy-17-epi-norethisterone were purchased from Toronto Research Chemicals (Toronto, Ontario, Canada). All other chemicals were of analytical grade and were purchased from Sigma Aldrich (Zwijndrecht, The Netherlands) unless stated otherwise.

### Enzymes and plasmids

A total of 83 CYP BM3 mutants were used. Detailed information about mutations present in the different mutants is tabulated in the Electronic Supplementary Material (ESM), Table S1. For 44 of the CYP BM3 mutants, construction has been described previously [14–20]. In total, 39 novel mutants were made by site-directed mutagenesis using the appropriate oligonucleotides (see ESM, Table S1). In general, mutations were introduced in the various templates in the pBluescript II KS(+) vector using the QuickChange Site-Directed Mutagenesis Kit (Stratagene). Forward primers that were used are mentioned in the ESM, Table S2. Reverse primers were exactly complementary to the forward primers. After mutagenesis, the presence of the desired mutations was confirmed by DNA sequencing (Service XS, Leiden, The Netherlands). Genes of the novel site-directed mutants were cloned from the pBluescript II KS(+) system, where they reside between the *BamHI/EcoRI* restriction sites, into the pET28a+ vector.

### Expression of CYP BM3 mutants

His-tagged pET28a+ constructs of wild-type (WT) CYP BM3 and all mutants were transformed in *Escherichia coli* BL21 (DE3) cells using standard procedures. For expression, 600 mL Terrific Broth (TB) [21] medium (24 g/L yeast extract, 12 g/L tryptone, 2 g/L peptone, 20 mL/L glycerol) with 30 μg/mL kanamycin was inoculated with 15 mL of an overnight culture. Cells were grown at 175 rpm and 37 °C until OD_600_ reached 0.6. Protein expression was induced by addition of 0.6 mM isopropyl-β-d-thiogalactopyrasonide (IPTG). Temperature was lowered to 20 °C and 0.5 mM of the heme precursor δ-aminolevulinic acid was added. Expression was allowed to proceed for 18 h. Cells were harvested by centrifugation (4600*×g*, 4 °C, 25 min), and the pellet was resuspended in 20 mL KPi-glycerol buffer (100 mM potassium phosphate (KPi) pH = 7.4, 10 % glycerol, 0.5 mM EDTA and 0.25 mM dithiothreitol). Cells were disrupted using an EmulsiFlex-C3 (Avestin Inc., Ottawa, Ontario, Canada; 1000 psi, 3 repeats), and the cytosolic fraction was separated from the membrane fraction by ultracentrifugation of the lysate (120,000*×g*, 4 °C, 60 min). CYP concentrations were determined using the carbon monoxide (CO) difference spectrum assay as described by Omura et al. [22].

### CYP BM3-mediated metabolism of the drugs amitriptyline and buspirone

Metabolic incubations for the drugs amitriptyline (AMI) and buspirone (BUS) were performed in 100 mM potassium phosphate (KPi) buffer at pH 7.4 with the cytosolic fraction containing 100 nM of BM3 mutant at a 40-μM substrate concentration. Final volume of the incubations was 250 μL, and the final DMSO concentration was always 5 %. Reactions were initiated by the addition of 50 μL of NADPH-regenerating system (NRS; final concentrations: 50 μM NADPH, 2.5 mM glucose-6-phosphate and 0.5 U/mL glucose-6-phosphate dehydrogenase). Reactions were allowed to proceed for 60 min at 24 °C and were terminated by addition of 250 μL ice-cold acetonitrile (ACN). Samples were subsequently centrifuged to remove precipitated protein (14,000 rpm for 15 min) and supernatants were analysed by LC-MS/MS.

Electrospray ionization LC-MS/MS analyses in the positive ion mode were carried out at the Division of Molecular Toxicology at the Vrije Universiteit in Amsterdam, The Netherlands. Metabolites and parent compounds were analyzed by reversed phase chromatography using a Synergi MAX-RP column (Synergi, 4 μm, 150 × 4.6 mm i.d.; Phenomenex, Amstelveen, The Netherlands) at a flow rate of 0.3 mL/min. The gradient was composed of solvent A (0.1 % *v*/*v* formic acid in water) and solvent B (0.1 % *v*/*v* formic acid in ACN). For the various substrates, different gradient programs were used. For identification of substrates and metabolites, a Finnigan LCQ Deca mass spectrometer (ThermoQuest-Finnigan) was used operating in the positive ion electrospray mode. N_2_ was used as a sheath gas (60 psi) and auxiliary gas (10 psi); the needle voltage was 5000 V and the heated capillary was at 150 °C. LC-MS/MS data of the substrates and bio transformation products were processed with Xcalibur/Qual Browser v 1.2 (ThermoQuest-Finnigan). Standard curves of the substrates were linear between 0.1 and 100 μM.

### Effects of co-administration of multiple drugs

To investigate effects of co-administration of multiple drugs, incubations were performed with CYP BM3 mutant MT72 (M11 V87F) using the marketed drugs AMI, BUS, coumarine (COU), clozapine (CLZ), dextromethorphan (DEX) and norethisterone (NET) in different combinations. All incubations had a final volume of 250 μL and consisted of KPi containing 250 nM of the cytosolic fraction of MT72 while the final DMSO concentrations were always of 5 %. The concentration of each individual drug was set at 100 μM. Reactions were initiated by addition of 50 μL NRS and were allowed to proceed for 60 min at 24 °C after which 750 μL ice-cold ACN was added. Samples were subsequently centrifuged to remove precipitated protein (14,000 rpm for 15 min) and supernatants were analysed by LC-MS.

LC-ESI-MS analyses were carried out at the Division of Molecular Toxicology at the Vrije Universiteit in Amsterdam, The Netherlands. Extracts were separated by reversed phase chromatography using a C18 column (Luna C18(2), 5 μm, 4.6 × 150 mm i.d.; Phenomenex, Amstelveen, The Netherlands) at a flow rate of 0.5 mL/min and a temperature of 25 °C. The gradient was composed of solvent A (0.1 % formic acid in water) and solvent B (0.1 % formic acid in ACN). The samples were analyzed on an Agilent 1200 Series Rapid resolution LC equipped with a Time-Of-Flight (TOF) Agilent 6230 mass spectrometer (Agilent technologies, Waldbronn, Germany). The electrospray interface was operated at a capillary voltage of 3500 V with N_2_ as drying gas (12 L/min) and nebulizer gas (pressure 60 psig). The gas temperature was 350 °C during operation. The TOF was used in the positive ion mode, and data was acquired using the Mass Hunter workstation software (version B.06.00). Standard curves of the substrates were linear between 0.1 and 100 μM.

### Screening of the CYP BM3 library using a cocktail approach

The degree of metabolism of six marketed drugs by the total CYP BM3 library was investigated using a cocktail approach. The six drugs used for this experiment were AMI, BUS, COU, DEX, diclofenac (DIC) and NET. The concentration of the six marketed drugs was set at 100 μM, the final DMSO concentration in the incubation was set at 5 % and all CYP BM3 mutants were incubated at a 250-nM enzyme concentration. Reactions were initiated by addition of 50 μL NRS and were allowed to proceed for 60 min at 24 °C. To terminate reactions, 250 μL of ice-cold ACN containing 50 μM minaprine was added after which samples were subsequently centrifuged to remove precipitated protein (14,000 rpm for 15 min). Each sample was analysed by UPLC-MS/MS while a selection of samples was also analysed by LC-MS.

UPLC-MS/MS analyses were carried out at QPS Netherlands B.V. in Groningen, The Netherlands. For UPLC-MS/MS, an AB SCIEX API 4000™ tandem MS System (AB SCIEX, Framingham, MA, USA) hyphenated with an Agilent 1290 Infinity Binary LC System (Agilent technologies, Waldbronn, Germany) was used. Electrospray ionization and a Multiple Reaction Monitoring (MRM) scan mode with a dwell time of 10 ms/s (see for more details the ESM, Table S3) was used. Instrument conditions were as follows: collision gas (CUR) 35, curtain gas (CAD), GS1 50, GS2 50, ion spray voltage 5500 and source temperature 550 °C. For sample pretreatment, 10 μL of supernatant from the incubations was mixed with 2000 μL mobile phase (90:10, A/B) to which 50 nM MIN was added. From this pretreated sample, 5 μL was injected for analysis. Separation was achieved with an Acquity UPLC BEH column (1.7 μm particles, 2.1 × 50 mm; Waters, Milford, USA) at a flow rate of 0.3 mL/min and a temperature of 40 °C. The data were processed with commercial available Analyst 1.4.1 (AB SCIEX) software. Standard calibration curves of all substrates and metabolites were linear between 0.1 and 100 μM.

LC-ESI-MS/MS analyses were carried out at the BioAnalytical Chemistry at the Vrije Universiteit in Amsterdam, The Netherlands. A Shimadzu (‘s Hertogenbosch, The Netherlands) ion-trap time-of-flight (IT-TOF) hybrid mass spectrometer equipped with a Shimadzu LC system (SIL-20 AC autoinjector, two LC-20AD pumps, CT-20AC column oven, SPD-AD UV/VIS detector and a CBM-20A controller) was used. The needle voltage was set to 4.5 kV, and the source heating block and the curved desolvation line were kept at 200 °C. A drying gas pressure of 62 kPa and a nebulizing gas (N_2_) flow rate of 1.5 L/min assisted the ionization. Full spectra were obtained in the positive ion mode between *m/z* 140 and 450. MS^2^ spectra were obtained in a data-dependent mode between *m/z* 100 and 450 with an ion accumulation time of 10 ms, a precursor width of 3 Da and a collision energy of 75 %. For sample pretreatment, 20 μL of supernatant from the incubations was mixed with 80 μL of 50 % ACN. From this pretreated sample, 12 μL was injected for analysis. Chromatography was performed on a Synergi MAX-RP column (Synergi, 4 μm, 150 × 4.6 mm i.d.; Phenomenex, Amstelveen, The Netherlands) at a flow rate of 0.4 mL/min and a temperature of 25 °C. The gradient was composed of solvent A (0.1 % formic acid in water) and solvent B (0.1 % formic acid in ACN). The data were processed with LCMS solution Version 3.60.361 (Shimadzu).

### Chemometric analyses

Raw data (.cdf files) resulting from LC-MS analyses of the CYP BM3 library screening using the cocktail approach (BM3-Cocktail data) were read into the R data analysis environment (R, 2.15, http://cran.r-project.org/) using the R ncdf package (version 1.6.6) and binned according to a *m/z* range of ±0.5 using the MassView package. The MassView package has been made in-house for binning mass data according to a specified *m/z* range and uses the caMassClass package version 1.9 (http://cran.rproject.org/src/contrib/Archive/caMassClass/). Data between 10 and 26.2 min were used for further analysis. Alignment of spectra was carried out with Parametric Time Warping (PTW) [23] using the R ptw package [24]. After zero padding (addition of 1000 zeros before and after each chromatogram for each *m/z*), PTW was executed twice using the mean chromatograms of the zero padded data and the aligned data, respectively, as reference. Resulting aligned data were converted in Matlab format using the R.matlab library 1.6.1 [25]. The size of the resulting data was 84 × 310 × 9720 (samples × *m/z* × time-points). For peak detection (for each nominal *m/z*), the method of Zhang et al. was applied [26]. The method is based on wavelets (haar wavelet, scale factor 60). Peaks (noise) below a value of 50,000 were not taken into account. Before peak detection, the chromatograms for each *m/z* were smoothed using a wavelet smoother (dog wavelet, Matlab cwtft function). Detected peaks with a signal-to-noise ratio below 2 (mostly chemical noise) were removed (noise calculated at the start- and endpoint of the peak using the data before smoothing). Detected peaks were integrated and stored in an array combined with associated *m/z* and time of peak maximum. During this procedure, corrections were made for small residual misalignment of peaks between the different samples (up to 3 s). To correct for sample processing variations and mass detector sensitivity changes, the data were normalised to the total mass of the first sample. Isotopes of substances were removed for *m/z* + 1, *m/z* + 2, *m/z* + 3 and *m/z* + 4 with additional constraints on the peak height ratios (roughly based on the natural ratios of ^1^H/D and Cl_35_/Cl_37_). Small differences in retention time were allowed (<15 s). Peaks related to substances present in the eluent, solvent and incubation solution and peaks of the substrates and the internal standard were removed using the peaks present in the WT sample. The resulting data is expected to comprise of the metabolites formed by enzymatic conversion of the substrates.

Outlier analysis was executed using Robust PCA [27]. Implementation in the Matlab-based LIBRA package was applied [28]. ROBPCA was applied using default settings. For retrieval of the contributing variables, the partial decomposition methodology of Alcale et al. [29] was implemented. For determination of the number of latent variables, the estimate factors function of the PLS toolbox was applied (PLS toolbox Version 7.0, Eigenvector Research, Inc., Manson USA). The principle of this function is that it selects those principal components which appear to be stable during resampling of the data. Clustering of data was obtained using hierarchical clustering [30]. Built-in Matlab procedures were used (Matlab version R2012b, Matlab, The MathWorks Inc., Natick, Massachusetts, USA). Besides these procedures and functions, several in-house functions were constructed to easily visualize subsets of the data. Functions were constructed to visualize *m/z* traces (chromatograms) for one specific *m/z* value for all samples and to visualize the identified peaks for a specific *m/z* and retention time combination in all samples.

## Results and discussion

### Selection of the CYP BM3 mutants

The aim of the present study is to investigate if a cocktail approach can be used to screen CYP BM3 libraries for metabolic activity and diversity. It was therefore decided to employ a CYP BM3 library consisting of mutants with as much structural diversity as possible by selecting mutants with mutations that preferably were introduced throughout the whole protein structure. In addition, CYP expression levels of the selected mutants needed to be suitable to allow large-scale enzyme expression. Mutants that were used in the current study were a combination of 44 mutants that have been described previously [14–20] complemented with 39 novel CYP BM3 mutants (see ESM, Table S1).

The set of 44 previously described mutants include M01, M02, M05 and M11 which were constructed by a combination of three site-directed mutations (R47L, F87V and L188Q) and subsequent rounds of random mutagenesis by error-prone PCR [31]. The 40 other mutants have all been based upon these four templates. This set of mutants has previously been tested against a range of substrates, including marketed drugs [14, 16, 32, 33], steroids [15, 18, 20], ionones [19], kinase inhibitors [34], alkoxyresorufins [15, 17] and endocrine disrupting chemicals [17, 35]. They metabolized these substrates with varying catalytic activities while also displaying significant differences in the metabolic profiles generated. The 39 mutants that have not been described previously were all created in-house by site-directed mutagenesis using the appropriate mutant templates (see ESM, Table S2). Mutations have been introduced at different positions throughout the protein in order to address, amongst others, effects upon regio- and stereo-selectivity (residues 72, 74, 75, 81, 82, 86, 87, 264, 328, 436, 437 and 440), organic solvent tolerability (residues 235, 471, 494 and 1024, see also [36]) and protein stability (residues 53, 176, 208 and 359). In-depth discussion of these properties is not a subject in this manuscript and will not be addressed.

Expression levels and stability of WT CYP BM3 and all 83 mutants were determined by measuring intensity of the characteristic Soret band at 450 nm upon reduction by dithionite and addition of CO. All cytosolic BM3 fractions were found to contain stable and active CYP protein. Although expression yields varied amongst the different mutants (data not shown), at least 50 nmols of active CYP protein was expressed per 600 mL of TB culture for each mutant and the WT enzyme. Cytosolic fractions were used to prepare 5 μM stock solutions in KPi-glycerol buffer for the WT enzyme and all mutants.

### Characterization of the CYP BM3 library-using amitriptyline and buspirone

**Fig. 1:**
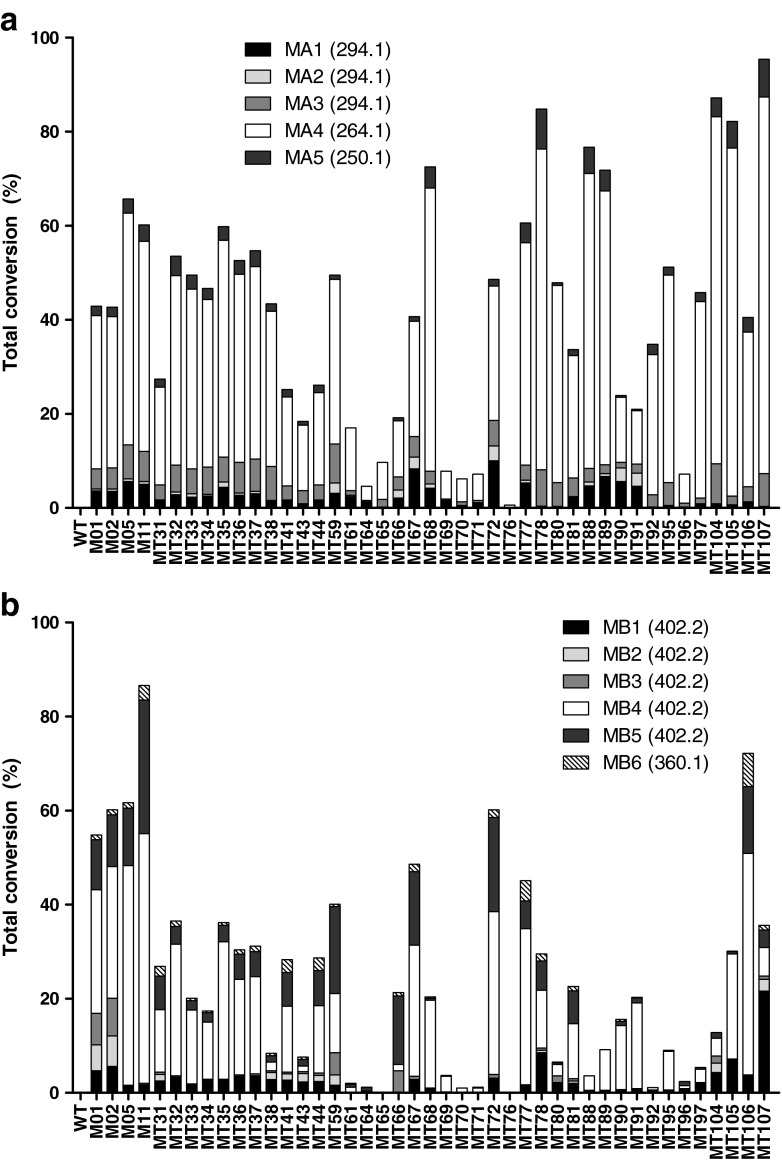
Product profile and activity of CYP BM3 engineered variants towards amitriptyline (**A**) and buspirone (**B**). Activities were determined by calculating the substrate depletion using the average peak area of the parent at 60 min and at time zero. Values represent the mean of two independent experiments with less than 10 % error. Product selectivity in % was calculated from the extracted ion chromatograms and is represented by *different shades* in each bar

In order to investigate the applicability of a cocktail approach to screen CYP BM3 libraries for metabolic activity and diversity, information is needed about activity of the mutants towards single substrates that are present in the cocktail. Since AMI and BUS have previously been successfully used to probe differences between CYP BM3 mutants, showing significant differences in metabolite profiles [16], it was decided to screen the WT enzyme and a selection of 43 mutants against both drugs. Figure 1a, b shows the diversity of the 44 tested enzymes towards AMI and BUS, respectively. As can be seen, both drugs are metabolized by almost all CYP BM3 mutants with varying activities into different metabolites.

For AMI (see Fig. 1a), the major product in all cases was nortriptyline (NOR; MA4) which is consistent with previous results [16]. Significant amounts of MA2 are only formed by a small number of mutants (MT35, MT59, MT66, MT67, MT68, MT72, MT90 and MT91). It is interesting to observe that all mutants that form MA2 contain a mutation at amino acid residue 87, residue 437 or mutations at both of these residues. MA1, MA3 and MA5 were formed by almost all mutants although at varying levels. The WT enzyme did not display any activity while MT76 only formed a very small amount of MA4. Identification of the metabolites of AMI is discussed in more detail in the ESM.

For BUS (see Fig. 1b), it was observed that significant differences existed between metabolite distribution profiles generated by the tested mutants. The WT enzyme, MT65 and MT76 did not display any activity towards BUS. MB4 (5-OH-BUS) was produced by all other mutants, although this was not the major metabolite in every incubation analysed. MB1 was not formed by MT64, MT65, MT66, MT70, MT71 and MT76, having in common that they contain a mutation at the 87 position (V87Q, V87E, V87G, V87K, V87M and V87W, respectively). When compared to their template M11, for MT70 and MT71, it appears that the mutation at position 87 resulted in a very large decrease of metabolic activity confirming that this position is crucial for efficient biotransformation of substrates [15]. MB2 was only formed by a selection of mutants (M01, M02, MT31, MT38, MT41, MT43, MT44, MT59, MT104 and MT107). The majority of the mutant library is derived from M11 (M11 + 28 M11-mutants; 66 %) while besides the WT enzyme, M02 and M05 the rest of the library is derived from M01 (M01 + 12 M01-mutants: 27 %). It is interesting to note that of the ten mutants that form MB2, only two are derived from M11 while the majority is derived from M01. These results suggest that the additional mutations in M11 compared to M01 and M02 (F81I, E143G, Y198C and H285Y) have an effect on the metabolism of BUS which is probably mainly caused by mutation of the active-site residue phenylalanine at position 81 into isoleucine. Significant amounts of MB3 were only produced by M01, M02, MT59, MT66 and MT80 while MB4 and MB5 were formed by almost all mutants although at varying levels. Identification of the metabolites of BUS is discussed in more detail in the ESM.

From all these data, it was again proven that the 87 position is crucial, and that significant differences in activity and metabolite profiles could be obtained with the mutant library.

### Validation of the cocktail screening approach

**Fig. 2:**
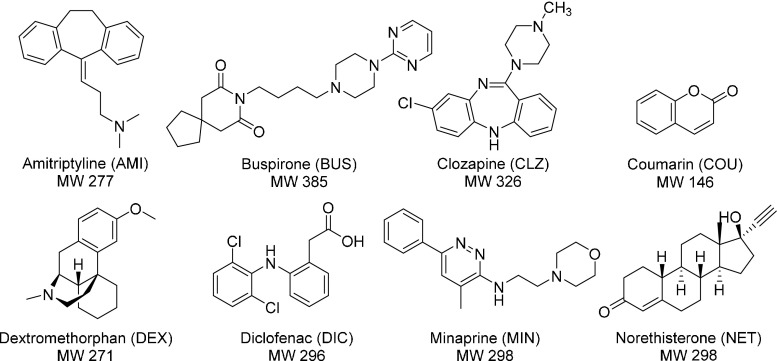
Structures of the marketed drug substrates that were used in this study. The molecular weight (MW) is indicated for each substrate

Having an established CYP BM3 mutant library, the next step in development of the cocktail screening was to investigate effects of co-administration of multiple drug substrates. Substrate mixes of various compositions were made, and effects of co-administration of multiple drugs on the metabolic activity and metabolic product profile of AMI and/or BUS by a single CYP BM3 mutant were investigated. The CYP BM3 mutant chosen for this experiment was MT72 since it displayed good activity towards both AMI and BUS during the previous single substrate screening experiment (see Fig. 1a, b). Besides AMI and BUS, the marketed drugs clozapine (CLZ), coumarin (COU), dextromethorphan (DEX) and norethisterone (NET) were selected for this experiment based on previous studies [20, 31, 32] (see Fig. 2 for chemical structures). Concentrations of the substrates were set at 100 μM which is higher than the concentrations used during the single substrate screening experiment (40 μM). In the latter experiment, a number of mutants displayed very high conversions (above 80 %) towards both AMI and BUS. It was therefore decided to increase all substrate concentrations to 100 μM.

**Fig. 3:**
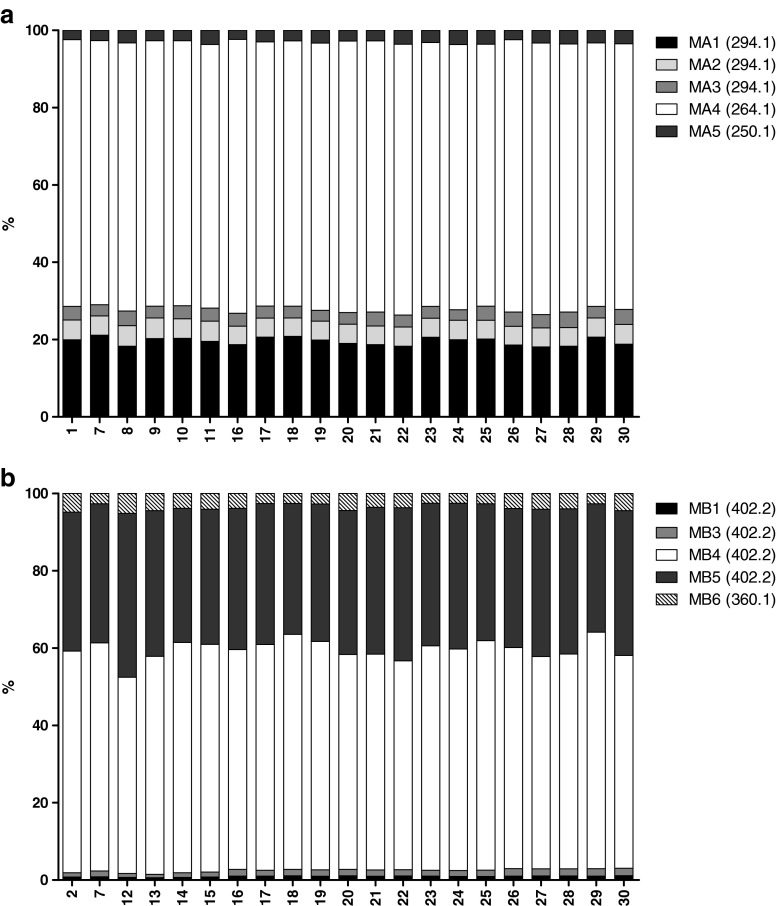
Effect of co-administration of multiple drugs on the metabolic profile of the drugs amitriptyline (**A**) and buspirone (**B**) generated by MT72. The sample numbers correspond to the incubations represented in Table 1

As can be seen from Table 1, substrate depletion was observed for all drugs screened in all incubations. When looking at the metabolic activity of MT72 towards AMI, it can be seen that the amount of AMI depletion varies between 34 and 64 % while the average AMI depletion in these incubations is 46.4 % with a standard deviation of 8.3 % (overall precision (CV) of 18 %). This amount of substrate depletion corresponds very well with the results from the single substrate screening experiment during which a depletion of 48.6 % was measured for MT72. There is no clear trend to observe in the effect of co-administration of the marketed drugs, as all drugs tested did not significantly induce or inhibit the metabolism of AMI and the number of drugs co-administered had no effect on the metabolic activity. Furthermore, it can be seen from Fig. 3a that the metabolite distribution is not affected by co-administration of multiple drugs. For AMI, the expected metabolites MA1-MA5 (based on the single substrate screening experiment) were formed while also the metabolic profile generated was similar as before (see Fig. 1a).

**Table 1:** Effect of co-administration of multiple drugs on metabolic activity of MT72

Sample	AMI (%)^a^	BUS (%)^a^	CLZ (%)^a^	COU (%)^a^	DEX (%)^a^	NET (%)^a^
1	49.3 ± 0.7	np	np	np	np	np
2	np	60.3 ± 1.3	np	np	np	np
3	np	np	4.0 ± 2.7	np	np	np
4	np	np	np	13.1 ± 3.08	np	np
5	np	np	np	np	7.3 ± 0.7	np
6	np	np	np	np	np	1.7 ± 1.0
7	37.7 ± 2.2	56.6 ± 2.8	np	np	np	np
8	56.5 ± 6.0	np	8.5 ± 2.4	np	np	np
9	51.6 ± 0.6	np	np	8.1 ± 1.9	np	np
10	35.5 ± 0.5	np	np	np	5.0 ± 0.2	np
11	64.0 ± 2.5	np	np	np	np	1.6 ± 0.12
12	np	69.5 ± 12.1	6.9 ± 3.9	np	np	np
13	np	76.2 ± 6.2	np	11.3 ± 1.7	np	np
14	np	61.1 ± 1.7	np	np	7.1 ± 0.5	np
15	np	60.2 ± 0.8	np	np	np	4.2 ± 0.5
16	34.6 ± 2.1	54.0 ± 2.1	2.0 ± 1.1	np	np	np
17	42.0 ± 3.6	61.4 ± 4.3	np	8.1 ± 1.6	np	np
18	37.3 ± 1.0	55.8 ± 1.4	np	np	2.2 ± 0.2	np
19	43.9 ± 4.5	60.2 ± 5.4	np	np	np	1.8 ± 1.0
20	41.2 ± 3.4	60.5 ± 4.6	8.4 ± 1.2	8.9 ± 0.5	np	np
21	43.5 ± 8.3	63.4 ± 11.9	8.1 ± 1.3	np	6.7 ± 0.4	np
22	51.8 ± 14.4	68.8 ± 18.1	9.6 ± 3.1	np	np	3.2 ± 2.9
23	47.8 ± 10.0	69.0 ± 13.9	np	7.4 ± 0.7	6.2 ± 0.5	np
24	51.6 ± 12.4	69.0 ± 15.6	np	3.2 ± 3.9	np	1.7 ± 0.9
25	46.5 ± 2.9	60.8 ± 4.3	np	np	6.5 ± 1.8	3.2 ± 0.9
26	35.9 ± 0.3	53.3 ± 0.6	4.2 ± 0.2	4.6 ± 0.2	3.7 ± 1.6	np
27	44.4 ± 3.5	58.3 ± 2.9	9.3 ± 1.7	9.2 ± 3.5	np	3.4 ± 2.6
28	45.5 ± 2.1	60.1 ± 2.5	6.0 ± 0.5	np	4.0 ± 2.4	2.7 ± 1.4
29	43.5 ± 1.5	56.4 ± 0.7	np	9.2 ± 2.0	6.2 ± 3.7	3.4 ± 2.8
30	51.6 ± 3.8	61.3 ± 3.8	15.0 ± 2.5	12.6 ± 6.7	8.6 ± 2.6	6.0 ± 4.7
Average	46.4 ± 8.3	61.7 ± 7.9	7.5 ± 3.8	8.7 ± 3.6	5.8 ± 2.2	3.0 ± 2.0

^a^ The substrate depletion is calculated by using the average peak area of the parent at 60 min and at time zero. Values are expressed in percentages of the average peak area of the parent at time zero. Measurements were performed in duplicate. ‘np’ indicates that the substrate was not present in the corresponding incubation

Considering the metabolic activity of MT72 towards BUS, it can be seen that the amount of BUS depletion varies between 53 and 77 % while the average BUS depletion in these incubations is 61.7 % with a standard deviation of 7.9 % (CV of 13 %). These findings correspond very well with the results from the single substrate screening in which a depletion of 60.2 % was measured. No significant alteration of the metabolite distribution occurs as result of the co-administration (see Fig. 3b). For BUS, the expected metabolites MB1, MB3, MB4, MB5 and MB6 (based on the single substrate screening experiment) were formed while also the metabolic profile generated was similar as before (see Fig. 1b).

For CLZ, DEX and NET, metabolic activity (see Table 1) and diversity (data not shown) were not affected by co-administration of multiple drugs. Metabolic activity was much lower than the activity observed for AMI and BUS (substrate depletion ranging between 3 and 9 %). CLZ was metabolized into two metabolites by MT72 which were identified as being clozapine *N*-oxide and *N*-desmethylclozapine, respectively. DEX was metabolized into a total of four products. Two products were a result of hydroxylation (MD3 and MD4) while the two other products were a result of demethylation (MD5 and MD6). NET was metabolized into one monohydroxylated product, MN1. Identification of the metabolites of CLZ, DEX and NET is discussed in more detail in the ESM.

For COU, substrate depletion was observed in all incubations. However, in none of the incubations, COU-related metabolites were detected. Besides the possibility that the absence of metabolites is caused by the simple fact that no biotransformation occurred, MS signals of metabolites might have been too low for detection caused by lack of optimal ionization conditions since MS signals of COU itself were also much lower (at least ten times) than the signals of the other drugs. Another possible explanation for the absence of COU-related metabolites is that COU might be metabolized into coumarin 3,4-epoxide (CE) which is a reactive intermediate that can react with proteins present in the incubation and could therefore not be detected. Subsequently, CE can be further metabolized into *o*-hydroxyphenylacetaldehyde (*o*-HPA). This metabolite has also not been detected in any of the incubations.

In conclusion, no inhibition of the biocatalytic activity of the CYP BM3 mutant MT72 for the applied substrates was observed. Neither did the molecular profiles of the metabolites change, indicating that the cocktail incubation is a valid approach for the generation of unique compound libraries and for the screening of unique biocatalytic properties of BM3 mutants.

### Screening of the CYP BM3 library using a cocktail approach

The next step of our study was to screen the complete CYP BM3 library encompassing the 83 mutants and the WT enzyme in a cocktail approach to investigate if such an approach can be used to rapidly classify mutant CYP BM3 libraries for both metabolic activity and diversity. The substrates that were selected for the cocktail screening experiment were AMI, BUS, COU, DEX, diclofenac (DIC) and NET. Besides one case report where a life-threatening dextromethorphan intoxication was associated with interaction with amitriptyline in a poor CYP2D6 metabolizer [37], no reports have been published about the interactions of any two or more of these drugs. Based on this information and the co-administration experiment described above, occurrence of drug interactions is not expected to have an impact. In order to rapidly assess bioactivity of the mutants, for each substrate, one of the previously described biotransformations was monitored. Based upon the single substrate screening experiment, NOR (MA4) and 5-OH-BUS (MB4) were selected for AMI and BUS, respectively. 7-OH-COU was selected as probe metabolite for COU. The demethylated products dextrorphan (MD5) and MM (MD6) were selected as probe metabolites for DEX whereas 4-OH-DIC was selected for DIC. For NET, 16β-hydroxy-17-epi-norethisterone was used as a probe metabolite. Standard calibration curves of all substrates and metabolites were obtained, and the limit of detection was below 0.1 μM in all cases. The results of the cocktail screening experiment are shown in Table 2.

**Table 2:** Metabolic activity of the CYP BM3 mutant library towards six drugs

Mutant	ΔAMI (μM) ^a^	NOR (μM) ^b^	ΔBUS (μM) ^a^	5-OH-BUS (μM) ^b^	ΔCOU (μM) ^a^	ΔDEX (μM) ^a^	MM (μM) ^b^	ΔDIC (μM) ^a^	OH-DIC (μM) ^b^	ΔNET (μM) ^a^
WT	3.5	0.2	6.3	0.1	21.2	<0 ^c^	0.1	9.3	0.1	25.7
M01	57.3	36.2	11.8	4.7	<0 ^c^	6.9	13.7	<0 ^c^	1.3	38.6
M02	57.2	44.1	16.4	4.8	17.6	3.2	17.2	7.1	1.0	56.6
M05	76.3	45.6	28.6	8.8	27.1	4.3	29.0	15.4	1.1	31.0
M11	79.2	49.1	29.67	10.0	19.5	6.2	29.8	13.0	2.4	29.0
MT21	6.9	1.1	11.9	0.1	5.2	<0 ^c^	0.2	7.3	0.1	21.1
MT22	3.0	0.4	6.4	0.1	0.8	<0 ^c^	0.2	6.2	0.1	35.7
MT24	6.3	0.9	15.2	0.1	27.5	<0 ^c^	0.2	13.8	0.1	18.4
MT28	30.2	18.0	21.2	7.5	1.7	<0 ^c^	5.6	1.3	1.0	17.4
MT30	41.9	32.0	7.6	2.4	7.6	2.8	9.4	<0 ^c^	0.5	<0 ^c^
MT31	42.0	31.4	5.2	1.7	<0 ^c^	7.9	8.9	<0 ^c^	0.7	<0 ^c^
MT32	68.3	41.6	<0 ^c^	2.0	<0 ^c^	21.4	20.6	9.9	9.4	19.3
MT33	67.2	39.7	10.8	2.1	7.6	15.0	17.8	11.2	7.9	7.0
MT34	61.1	41.2	5.2	1.4	<0 ^c^	9.2	14.2	3.3	2.8	8.7
MT35	77.7	46.3	15.3	5.8	3.2	20.4	24.2	4.3	4.5	4.4
MT36	62.3	38.2	6.9	1.2	<0 ^c^	13.3	12.6	<0 ^c^	1.9	16.3
MT37	70.2	39.3	8.6	1.9	<0 ^c^	10.4	16.1	3.0	2.9	16.3
MT38	55.1	40.4	6.3	0.3	<0 ^c^	12.7	16.7	0.5	0.3	6.9
MT39	1.9	0.6	9.4	0.1	5.4	3.6	0.3	2.3	0.1	0.5
MT40	5.4	0.5	1.3	0.1	8.4	<0 ^c^	0.3	1.2	0.1	1.8
MT41	46.8	30.7	13.1	3.0	<0 ^c^	10.0	8.5	<0 ^c^	0.6	2.7
MT42	68.7	51.9	4.4	0.6	<0 ^c^	18.2	16.4	<0 ^c^	0.7	<0 ^c^
MT43	32.2	20.3	14.2	0.2	3.2	4.3	6.1	<0 ^c^	0.1	<0 ^c^
MT44	44.0	30.0	7.2	2.3	7.5	15.8	7.9	<0 ^c^	0.5	6.1
MT45	3.6	8.3	7.4	0.1	3.0	2.0	0.9	<0 ^c^	0.5	<0 ^c^
MT46	34.4	24.1	13.2	3.3	<0 ^c^	4.0	1.9	<0 ^c^	0.5	<0 ^c^
MT47	33.0	32.7	6.7	5.4	14.3	0.4	2.3	4.0	0.1	<0 ^c^
MT48	57.5	31.6	1.2	0.3	5.1	0.1	2.0	<0 ^c^	0.2	0.2
MT59	49.8	42.1	22.5	3.1	<0 ^c^	38.4	49.6	0.9	1.1	35.5
MT61	25.4	28.1	7.9	1.1	4.6	5.9	10.0	<0 ^c^	0.5	2.1
MT64	8.4	7.7	6.1	0.6	4.6	4.5	3.3	1.5	1.0	0.6
MT65	31.5	27.1	3.2	0.2	<0 ^c^	10.6	12.6	<0 ^c^	0.1	<0 ^c^
MT66	25.2	14.2	25.3	0.4	0.5	50.6	31.9	2.9	0.1	13.5
MT67	22.9	19.4	36.6	10.2	10.9	4.3	4.0	13.1	35.5	<0 ^c^
MT68	64.9	50.9	17.5	10.0	1.6	23.6	32.4	3.7	5.0	6.0
MT69	3.7	16.7	14.6	9.6	12.4	<0 ^c^	1.9	10.3	5.5	9.1
MT70	13.9	6.4	3.6	2.9	5.4	<0 ^c^	0.7	<0 ^c^	0.1	<0 ^c^
MT71	29.4	4.7	4.7	0.7	<0 ^c^	12.0	2.2	5.6	1.0	1.6
MT72	33.4	23.7	52.3	14.5	18.9	4.7	5.3	19.6	38.7	8.2
MT76	<0 ^c^	1.0	2.3	0.1	7.2	2.5	0.7	<0 ^c^	0.1	<0 ^c^
MT77	27.5	28.3	9.9	4.9	4.2	3.3	15.8	7.1	2.1	11.3
MT78	55.4	48.2	12.6	0.9	18.6	17.3	25.5	5.9	0.8	16.5
MT79	74.1	37.1	11.4	9.9	<0 ^c^	20.3	20.5	<0 ^c^	2.4	<0 ^c^
MT80	42.6	55.1	2.6	1.0	<0 ^c^	22.7	23.8	<0 ^c^	0.1	1.8
MT81	52.5	31.7	9.8	2.1	10.2	15.7	13.7	5.5	1.6	6.5
MT83	13.6	0.5	4.6	0.1	6.0	3.0	0.3	6.3	0.1	1.0
MT86	6.2	0.4	14.0	0.5	<0 ^c^	<0 ^c^	0.2	8.9	0.1	0.1
MT87	5.4	0.4	23.9	0.1	3.3	0.5	0.2	0.8	0.1	5.2
MT88	77.5	35.1	7.6	1.2	11.1	28.1	35.4	20.5	21.3	15.3
MT89	70.1	34.5	0.8	3.7	<0 ^c^	40.0	41.0	<0 ^c^	8.6	<0 ^c^
MT90	17.8	7.4	13.7	6.8	1.9	0.6	2.3	10.9	27.6	2.5
MT91	20.4	18.5	37.3	22.2	0.5	3.3	4.3	10.2	17.1	1.7
MT92	23.6	20.3	4.4	0.2	5.5	<0 ^c^	5.1	8.6	1.1	12.0
MT94	27.6	18.1	39.5	0.7	<0 ^c^	4.6	5.1	7.2	7.8	10.5
MT95	53.6	54.3	9.0	0.4	9.4	<0 ^c^	3.9	4.3	0.1	7.4
MT96	43.1	7.1	4.4	0.5	<0 ^c^	17.4	2.1	6.1	0.8	<0 ^c^
MT97	21.3	29.9	0.4	0.9	<0 ^c^	3.9	5.6	1.0	0.5	<0 ^c^
MT99	68.9	49.7	16.7	8.5	4.0	21.5	14.5	<0 ^c^	2.2	3.9
MT100	32.4	42.1	14.47	6.5	<0 ^c^	<0 ^c^	15.1	3.8	1.5	11.6
MT101	0.6	4.2	3.8	1.6	<0 ^c^	7.0	2.4	<0 ^c^	1.8	5.6
MT102	2.1	1.5	3.1	0.2	<0 ^c^	<0 ^c^	0.9	6.2	0.1	81.6
MT103	40.6	33.9	7.0	0.6	<0 ^c^	8.2	5.9	<0 ^c^	0.1	47.8
MT104	67.6	60.7	14.5	0.7	15.7	27.8	34.4	10.2	0.1	49.4
MT105	71.5	65.5	14.2	7.3	<0 ^c^	46.6	46.8	<0 ^c^	1.8	<0 ^c^
MT106	37.7	30.5	9.3	2.1	11.9	0.9	5.3	4.3	3.6	2.1
MT107	77.7	69.3	5.3	0.7	17.4	13.0	19.0	<0 ^c^	0.4	25.4
MT108	46.5	35.7	41.5	0.3	<0 ^c^	17.0	19.6	<0 ^c^	0.1	10.0
MT110	1.4	0.7	2.6	0.5	<0 ^c^	2.0	0.6	<0 ^c^	0.1	4.5
MT111	19.7	16.7	6.6	1.7	6.2	5.7	5.2	<0 ^c^	0.5	<0 ^c^
MT112	33.8	13.1	58.8	1.3	13.3	4.9	5.0	<0 ^c^	0.5	<0 ^c^
MT113	27.9	25.7	5.9	2.1	<0 ^c^	6.0	9.9	3.2	0.4	2.9
MT114	46.7	33.5	66.6	2.4	8.6	14.2	17.2	<0 ^c^	1.0	<0 ^c^
MT120	27.1	29.7	7.5	0.3	<0 ^c^	<0 ^c^	4.1	2.4	1.3	12.8
MT121	66.1	55.5	14.1	1.7	<0 ^c^	<0 ^c^	3.1	<0 ^c^	2.5	<0 ^c^
MT122	53.8	39.2	7.9	4.1	<0 ^c^	17.3	21.0	<0 ^c^	0.8	<0 ^c^
MT124	41.3	39.6	1.1	0.5	<0 ^c^	6.4	8.6	<0 ^c^	0.9	<0 ^c^
MT125	42.0	40.7	<0 ^c^	0.6	<0 ^c^	<0 ^c^	6.8	<0 ^c^	0.3	83.4
MT126	81.7	77.6	6.2	0.7	2.9	19.4	28.7	<0 ^c^	0.2	3.2
MT127	73.5	59.5	14.7	1.9	9.6	30.1	34.8	11.2	3.1	15.7
MT128	12.1	8.9	−0.6	0.4	0.7	7.0	4.7	<0 ^c^	0.1	<0 ^c^
MT129	64.2	29.4	56.3	0.9	<0 ^c^	37.9	38.6	0.2	0.6	3.3
MT130	3.7	5.9	<0 ^c^	0.2	<0 ^c^	8.5	3.5	<0 ^c^	0.1	<0 ^c^
MT131	31.2	20.5	10.4	2.2	<0 ^c^	<0 ^c^	4.0	4.1	0.5	15.8
MT132	10.9	11.5	5.1	0.6	1.7	17.5	14.8	<0 ^c^	0.1	7.3
Mutant	ΔAMI (μM)	NOR (μM)	ΔBUS (μM)	5-OH-BUS (μM)	ΔCOU (μM)	ΔDEX (μM)	MM (μM)	ΔDIC (μM)	OH-DIC (μM)	ΔNET (μM)

^a^ The substrate depletion is calculated by using the average peak area of the parent at 60 min and at time zero. Values are expressed as μM substrate and measurements were performed in triplicate. The substrates used are amitriptyline (AMI), buspirone (BUS), coumarine (COU), dextromethorphan (DEX), diclofenac (DIC) and norethisterone (NET)

^b^ The amount of product formed is calculated by using the average peak area of the metabolite at 60 min. Measurements were performed in triplicate. The metabolites screened for were nortriptyline (NOR), 5-hydroxybuspirone (5-OH-BUS), 3-methoxymorphinan (MM) and 4′-hydroxydiclofenac (OH-DIC)

^c^ A negative substrate depletion was measured which indicates that the averaged substrate concentration of the parent drug was higher in the incubations at 60 min compared to the incubations at time zero

It can be seen that significant differences exist between metabolic activities of the different mutants towards the six drugs. The averaged standard deviation of the substrate depletions calculated were below 15 % for all substrates (see also ESM, Table S4). Averaged standard deviation of the metabolite concentrations determined were 3.0, 0.3, 1.0 and 0.4 μM for NOR, 5-OH-BUS, MM and OH-DIC, respectively. For AMI, detection of the metabolite NOR corresponded well with the amount of substrate depletion observed. This is in agreement with the previous finding that NOR was the major metabolite during the single substrate screening experiment. For BUS, the substrate depletion determined was in most cases significantly higher than the amount of 5-OH-BUS detected. This indicates that more metabolites are formed which agrees with the previous results of the single substrate screening experiments. For COU, significant amounts of substrate depletion were observed with MT24 being the most active mutant. However, no formation of 7-OH-COU was detected. For DEX, formation of the *N*-demethylated product MM was detected while formation of the *O*-demethylated product dextrorphan was not detected. When comparing the amount of substrate depletion with the concentration of metabolite detected, it can be seen that in many cases the concentration of MM formed is higher than the amount of DEX which has been consumed. In addition, for several mutants, a negative amount of substrate depletion was observed. A possible explanation for these effects is, again, that factors present (including formed metabolites) in the incubation mixture may influence the MS signals of DEX and MM. For DIC, formation of 4-OH-DIC was detected, and in most cases, the amount of substrate depletion observed was higher than the concentration of 4-OH-DIC detected. This could indicate that other products are formed and agrees with the previous finding that DIC can be converted by CYP BM3 mutants into reactive metabolites [38], although in the respective study the amount of GSH-conjugates formed by most mutants did not account for more than 10 % of the total amount of metabolites produced. For NET, significant differences in the amounts of substrate depletion were determined for the tested mutants. However, formation of 16β-hydroxy-17-epi-norethisterone was not detected in any of the incubations. For a number of mutants, a negative amount of substrate depletion was observed.

Selected incubations were qualitatively analyzed by LC-MS/MS on a Shimadzu IT-TOF in order to provide accurate mass information on the substrates, metabolites and their fragment ions and thereby get more information about the metabolic profiles generated by the different mutants for the six drugs tested. For each mutant, one sample of the incubation at 60 min was analyzed while for a selection of mutants also an incubation at time zero was analyzed.

### Interpretation of the cocktail screening data

In order to scan the data in depth for known, but also unknown metabolites, several questions can be asked and several strategies can be followed. Regarding the questions, one can try, e.g. to: (1) look for all metabolites that are produced, (2) look for mutants that uniquely produce certain metabolites, (3) look for the most active mutants, (4) relate groups of enzyme mutations to certain metabolic processes, or (5) look for mutations which more efficiently produce certain metabolites. With respect to the strategies, one can (a) manually scan the data for all theoretical possible metabolic conversions (and combinations of conversions) for all substrates and (b) use certain procedures and techniques which are more specifically targeted at the different questions. The former strategy is a rather laborious strategy, especially if multiple substrates need to be investigated in combination with large numbers of mutant enzymes. In this case, an untargeted approach provides a better alternative. There are several simple data visualization and analytical techniques available to facilitate screening and investigation of data resulting from experiments in view of the various afore mentioned questions. First of all, raw LC-MS data files need to be pre-processed. Katajamaa and Orešič [39] and Draper et al. [40] describe the possible steps in the data (pre)processing (processing pipeline). In this work, we have followed a slightly different order. Firstly, data were aligned using PTW, followed by a peak detection procedure, as described in the ‘Materials and Methods’ section. The result of the application of such a processing pipeline is a reduced data set containing only peaks (including peak area or height, position in time and *m/z* dimension) of substances which could be of possible interest.

One of the most basic methods to obtain a visual overview of such multidimensional data is PCA, which can be combined with information on the most important variables (peaks) using a biplot [41]. PCA focusses on the variation in data. Frequently, this variation is due to metabolites that are produced in high quantities by most enzymes (cf. questions 1 and 3). If one wants to focus on those mutants that are specifically producing one or a few metabolites (cf. question 2), these cannot readily be identified using PCA and therefore another approach needs to be applied. Robust PCA is a methodology specifically constructed for identifying potential outliers in data sets. Selected mutant enzymes that produce specific metabolites differ from most other enzymes and thus can be viewed as being outliers. Moreover, clustering techniques can be applied to facilitate recognition of certain groupings in the data being either the detected peaks or the types and sites of mutations. For a comparison between the various chemometric analyses and manual data analysis, we have also manually screened all IT-TOF MS data files upon the presence of CYP-related metabolites. For quantification of the parents and metabolites, peak areas from the MS spectra were assumed to yield similar MS responses, although it is realized that MS signals generated by the different metabolites in most cases are not similar to those generated by the parent compound. However, as no UV data is available, quantification based upon the MS signal is the only alternative in order to generate metabolite profiles. Detailed results of the qualitative analysis of the metabolism of AMI, BUS, DEX, DIC and NET can be found in the ESM (Figures S1-S5).

**Fig. 4:**
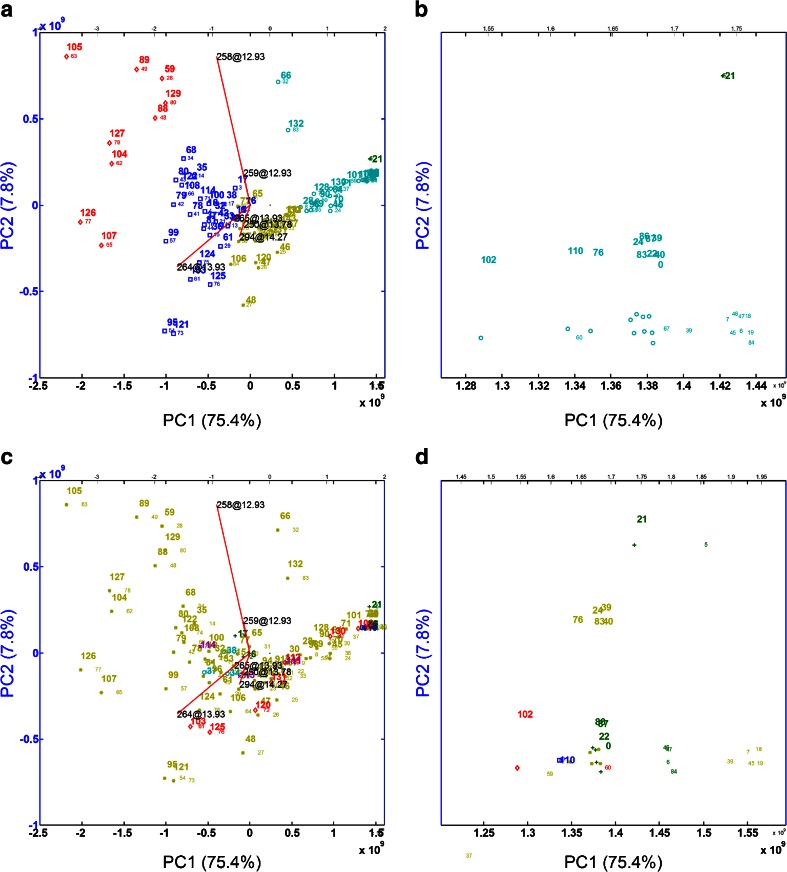
Biplot of the pre-processed data. The samples are identified with the MT-number, the peaks indicated by lines and labelled with the corresponding *m/z* and retention time. The *small numbers* indicate the order of samples in the data set. 0 (84) indicates the WT. (**A**) The data are coloured according to a hierarchical clustering (average linkage) of the data based on 5 clusters. In order to prevent that too many peaks are plotted a user definable threshold of mostly 10 % of the largest peak was used; (**B**) zoomed-in version of the central right part of (**A**); (**C**) biplot of the pre-processed data with colour coding according to the clustering of the mutation patterns of the mutants (using hierarchical clustering, average linkage) based on 6 clusters; (**D**) zoomed-in version of the central right part of (**c**)

The result of the application of PCA on pre-processed data from which substrates and their isotopes are removed is visible in Fig. 4a. This is a so-called biplot in which samples are presented together with (in this case a subset of) peaks mainly responsible for the spread in the data. From right to left, there is an increase in metabolic production. A zoomed-in version of the samples on the right is visible in Fig. 4b. Clearly, the samples can be identified that hardly or not display any significant activity or metabolite formation for any of the drugs tested.

The data describing the mutations present in the CYP BM3 library (with zeros and ones, the latter indicating that a certain mutation is present in a mutant) can also be subjected to clustering. Based on the so-called cityblock distance and using average linkage hierarchical clustering, the data was clustered in 6/8 clusters: 5/7 small clusters and one large cluster. If the mutants in Fig. 4a are coloured according to this clustering, this results in Fig. 4c, d. This plot does not reveal much information, apart from one striking feature: the mutants belonging to the dark green cluster are located either on the right side of the figure (MT21, MT22, MT87, MT88; MT0 is the WT; see Fig. 4d), indicating that they do not produce any metabolite, or in the centre of the figure (MT16 and MT17). All these mutants contain only one to four mutations. The latter two mutants only contain the mutation A964V and G1049E, respectively, indicating that these mutations could be important for the production of metabolites.

In Fig. 4a, c, the main metabolites of the different substrates can be tentatively identified on basis of the mass spectrometric data. Several of the peaks in this plot are metabolites of AMI. The peak at *m/z* 264@13.93 (and its isotope at *m/z* 265@13.93) is the major metabolite MA4 (NOR). The peak at *m/z* 294@14.27 corresponds with MA3, while peak at *m/z* 250@13.78, corresponding with MA5, is also formed by most mutants. That they appear in this plot indicates that they explain the largest variation between the samples and that they are probably present in a large number of samples in high concentration. This is confirmed by Fig. 1a and ESM Fig S1. By checking the mass trace of *m/z* 294, more metabolites can be identified (MA1 at 12.3 min and MA2 at 12.8 min; see ESM Fig S6). By checking the corresponding mutants, it can be concluded that MA2 is formed mainly by mutants containing a mutation at position 87, position 437 or mutations at both of these positions which is in agreement with the results from the single substrate screening experiment.

The largest peak in Fig. 4a is the result of a DEX metabolite (see below for more details). When this DEX metabolite and the AMI metabolite MA4 are removed from the data, three metabolites of BUS appear in the biplot (data not shown). The most prominent peak at *m/z* 402@12.39 corresponds to MB4 (5-OH-BUS) while the peaks at *m/z* 402@12.87 and at *m/z* 360@12.14 correspond to MB5 and MB6, respectively. If the mass trace of *m/z* 402 is plotted (ESM Fig S7), five different metabolites appear in different quantities. Almost all mutants form MB4 (5-OH-BUS) while MB2 is only formed by a limited number of mutants. This is in agreement with the results from the single substrate screening experiment. For formation of MB2, again, the trend is observed that the majority of the mutants that form significant amounts of this metabolite do not contain the F81I mutations. The fact that mutant MT122, which is a mutant of M01 where the F81I mutation was introduced, does not form MB2 supports this hypothesis. In Table 3, a comparison has been made between the results of the screens for metabolic activity and diversity for a selection of mutants towards AMI and BUS. It can be seen from Table 3 that differences exist between the results from the different screenings. The substrate depletions calculated based on the results of the qualitative analysis by IT-TOF are in almost all cases (except for MT68-mediated conversion of BUS) lower than the substrate depletion calculated from the quantitative analysis by UPLC-MS/MS. This can be caused by the fact that MS signals generated by the different metabolites are not similar to those generated by the parent compound. The discrepancy between the percentage of depletion and the amount of metabolite quantified by UPLC-MS/MS can in most cases be explained by the formation of additional metabolites. This is especially the case for the MT66-mediated metabolism of BUS where the detected 5-OH-BUS (MB4) is only detected as a minor metabolite by IT-TOF. Trends of the metabolic activities and diversities measured by LCQ during the single substrate screening experiment and by IT-TOF during the cocktail screen correspond well. Mutants that displayed good activity during the single substrate screen also displayed good activity during the cocktail screen and metabolic profiles were very similar. This again demonstrates that co-administration of multiple drugs in a single incubation has no major effects on the metabolic activity and diversity of the tested mutants.

**Table 3:** Metabolic activity and diversity of selected CYP BM3 mutants towards amitriptyline and buspirone

AMI Mutant	UPLC (%) ^a^	UPLC NOR (μM) ^b^	IT-TOF (%) ^c^	MA1 %	MA2 %	MA3 %	MA4 %	MA5 %		LCQ (%) ^d^	MA1 %	MA2 %	MA3 %	MA4 %	MA5 %	
M11	79.2 ± 9.3	49.1 ± 0.8	58.9	19	1	22	50	8		60.2	9	1	10	74	6	
MT107	77.7 ± 4.4	69.3 ± 2.5	60.5	1	–	13	78	8		95.3	1	–	2	90	7	
MT35	77.7 ± 4.5	46.3 ± 2.2	56.5	17	4	17	56	6		59.8	7	2	9	77	5	
MT88	77.5 ± 11.6	35.1 ± 11.2	46.4	17	1	6	69	7		76.7	6	1	4	82	7	
M05	76.3 ± 8.1	45.6 ± 8.1	57.7	25	2	23	46	4		65.7	9	1	11	75	4	
MT105	74.1 ± 2.0	65.5 ± 5.7	52.6	2	–	5	85	8		82.1	1	–	10	85	4	
MT37	73.5 ± 5.8	39.3 ± 3.7	50.9	16	2	22	54	6		54.7	5	1	13	75	6	
MT89	71.5 ± 5.8	34.5 ± 9.5	45.4	21	2	5	67	5		71.7	9	1	3	81	6	
BUS Mutant	UPLC (%) ^a^	UPLC 5-OH-BUS (μM) ^b^	IT-TOF (%) ^c^	MB1 %	MB2 %	MB3 %	MB4 %	MB5 %	MB6 %	LCQ (%) ^d^	MB1 %	MB2 %	MB3 %	MB4 %	MB5 %	MB6 %
MT72	52.3 ± 4.4	14.5 ± 0.7	38.5	12	3	7	42	23	13	60.2	5	–	1	58	33	3
MT91	37.3 ± 20.4	22.2 ± 1.2	34.8	13	–	1	72	6	8	20.3	4	–	–	89	5	2
MT67	36.6 ± 5.6	10.2 ± 1.3	33.5	11	2	6	46	17	18	48.5	6	–	1	58	32	3
M11	29.6 ± 5.7	10.0 ± 0.4	26.8	8	1	2	57	13	19	86.6	2	–	–	61	33	4
M05	28.6 ± 7.9	8.8 ± 0.6	21.9	10	3	1	69	8	9	61.8	2	–	–	76	20	2
MT66	25.3 ± 4.0	0.4 ± 0.1	24.2	2	3	47	6	34	8	21.3	–	–	22	6	69	3
MT59	22.5 ± 3.6	10.2 ± 0.3	24.9	7	9	28	27	25	4	40.1	4	6	12	32	45	1
MT68	17.5 ± 10.7	10.0 ± 1.1	18.4	8	–	–	89	–	3	20.4	5	–	–	92	2	1

^a^ The substrate depletion is calculated by using the average peak area of the parent at 60 min and at time zero. Values are expressed in percentages of the average peak area of the parent at time zero. Measurements were performed in triplicate and results were obtained during analysis of the cocktail incubations by UPLC-MS/MS

^b^ The amount of product formed is calculated by using the average peak area of the metabolite at 60 min. Measurements were performed in triplicate and results were obtained during analysis of the cocktail incubations by UPLC-MS/MS

^c^ The substrate depletion is calculated by using the peak area from the MS signal of the parent at 60 min and the sum of the peak areas from the MS signals of the parent and its metabolites at 60 min. The substrate depletion is expressed as percentage of parent that has been converted into metabolites. Results were obtained during analysis of the cocktail incubation by LC-MS/MS on the IT-TOF

^d^ Activities were determined by calculating the substrate depletion using the average peak area of the parent at 60 min and at time zero. Values represent the mean of two independent experiments with less than 10 % error. Product selectivity in % was calculated from the extracted ion chromatograms. Results were obtained during analysis of the single substrate incubations by LC-MS/MS on the LCQ

The largest peak in Fig. 4a appears to be the *N*-demethylated product MM (MD6) (and parallel with it its *m/z* + 1 isotope) of DEX. When plotting the mass traces of *m/z* 258 (data not shown), it was found that the majority of the mutants produced the *N*-demethylated product MM (MD6) as the major product (see also ESM, Fig S3 and Table S5). Very small amounts of the *O*-demethylated product dextrorphan were also seen in this plot (at 11.55 min). These were formed by a selection of mutants (MT34, MT35, MT36, MT37, MT38 and MT42) which contained mutations at active-site residues S72, A74 or L437. However, also for these mutants, MM was the major product.

**Fig. 5:**
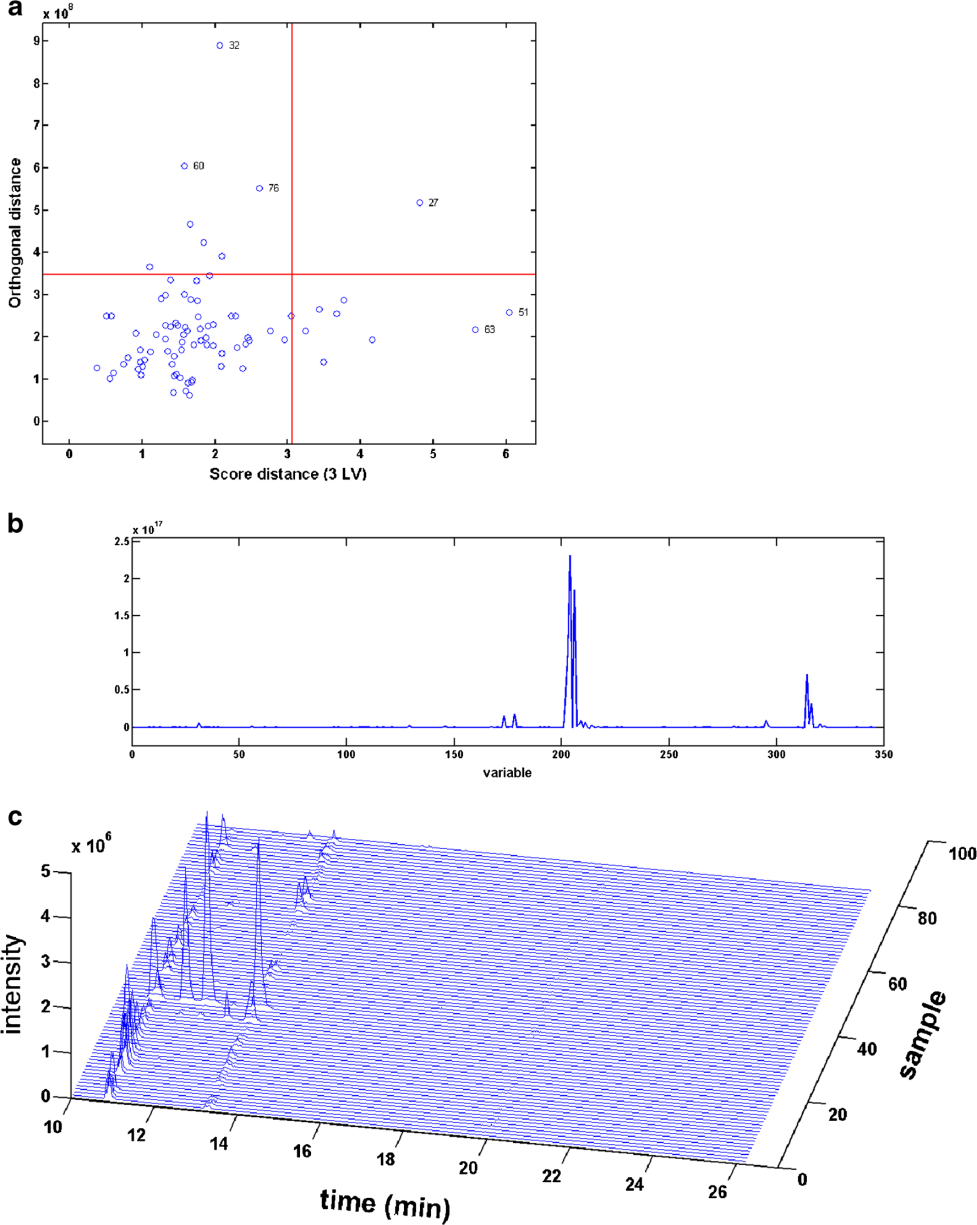
(**A**) ROBPCA plot of data set. Samples positioned in the *upper left quadrant* are called orthogonal outliers. Also samples in the upper right quadrant can be investigated; (**B**) a plot of the variables responsible for the outlying behaviour of sample 32 = MT66; the variable numbers in this plot are linked to chromatographic peaks at specific *m/z* – RT combinations; (**C**) a mass trace of *m/z* 288 showing the five hydroxylated DEX metabolites MD1-MD5; the sample numbers indicate the order of the samples in the data set. In the biplot in Fig. 4a, they are linked to the mutant (MT) numbers, used in other figures and tables

By using biplots, either directly or after removing the main peaks, the prominent metabolites of the various substrates can be retrieved (satisfying goal 1 and 5 when combined with mass traces or plots of the selected peaks for all mutants). Most of the times, the mutants that lie in the direction of such a peak produce high amounts of that specific metabolite. The procedure of removing the main peaks from the data, reconstructing and analysing the biplot, can be repeated, thereby retrieving also less prominent metabolites. Another approach is the ROBPCA-based outlier detection procedure. When applied on the data, it appeared that sample 32, which corresponds with MT66, was an outlying sample (see Fig. 5a). In Fig. 5b, the variables are plotted which are responsible for this outlying behaviour. The peaks as variable 202–204 and 206 appear to be peaks with a *m/z* value of 288 (the adjacent small peaks are their M + 1 isotopes), being four hydroxylated DEX metabolites which almost uniquely and in large amounts are produced by MT66 which displayed the highest activity towards DEX (see Fig. 5c).

UPLC overall has shown that a cocktail approach can be used to screen CYP BM3 mutants for metabolic activity and diversity. Different approaches have been used to assess the activities of the mutants. On the one hand, a UPLC-MS/MS-based method was used that measured depletion of the parent drugs and additionally monitored formation of at least one probe metabolite for each drug. This method made use of very short gradients (<5 min) and can be used to screen large amounts of samples in a relatively short time. The results of the UPLC-MS/MS-based screening were used to calculate substrate depletions for the tested drugs by the mutant library which, in most cases, provided reliable information about the activities of the mutants towards the tested drugs (except for COU). Inclusion of at least one probe metabolite for each drug makes the screening assay more reliable since it will make it easier to identify false positives when it is known which metabolite is expected to be formed. However, this is of course not always possible, especially not when the purpose of the screening is to identify novel mutants that can create chemical diversity by forming novel metabolites. Additional qualitative analysis of selected samples by an information-rich analysis method such as LC-MS/MS proved to be very valuable during this study as it provided much needed information about the metabolic profiles generated by the different mutants. The results of the screening suggest that the majority of the CYPs screened displayed a metabolic profile that is most similar to CYP3A4 (see ESM for more details). The chemometrical analysis facilitated an easy visualization and screening of the resulting data and a quick retrieval of the metabolites of the substrate cocktail. This appeared to be less laborious and time-consuming than manual analysis of the data.

## Conclusions

With the experimental setup chosen, significant differences in the drug-metabolizing potential were encountered for the CYP BM3 mutants tested and large differences in the biotransformation profiles generated were established. Therefore, it can be concluded and proven based on the data presented that the proposed analytical strategy is valid, and that we successfully developed a LC-MS/MS-based cocktail screening method for the classification of CYP BM3 mutant libraries for metabolic activity in a quantitative assay method and diversity in a qualitative assay method. By combining all data, the classification of the mutants was made feasible using well-defined chemometrical methods for the rapid and valuable mining of the data. Hence, the classification is of crucial importance to select the proper mutants to generate predesigned compound libraries rather than screening all mutants for each sort of biotransformation. The mutants described in this study could be useful as a starting point for further site-directed or random mutagenesis, to further enhance metabolic efficiency and change substrate diversity and regioselectivity. More research to rationalize the effects of the introduced mutations would be very valuable in this process. In the current study, the presented strategy was used to evaluate the metabolic efficiency of a library of CYP BM3 mutants towards in total six drugs. The analytical approach and designed experiments described in this article will form the basis for future research in screening for CYP BM3 mutants with improved metabolic activity and diversity. This will aid to further improve lead diversification in the drug discovery process and biosynthesis of drug(like) metabolites.

## Electronic supplementary material

ESM 1 (PDF 369 kb)
