# Supplementary material for: Application of a cocktail approach to screen cytochrome P450 BM3 libraries for metabolic activity and diversity
Source: Anal Bioanal Chem. 2016 Jan 11;408:1425–43. doi: 10.1007/s00216-015-9241-x (PMC4723632; doi:10.1007/s00216-015-9241-x)
Supplement: Supplementary file 1 — (PDF 369 kb) [file 216_2015_9241_MOESM1_ESM.pdf]

Analytical and Bioanalytical Chemistry

Electronic Supplementary Material

Application of a cocktail approach to screen Cytochrome P450 BM3 libraries for metabolic activity and diversity

Jelle Reinen, Geert Postma, Cornelis Tump, Tom Bloemberg, Jasper Engel, Nico P. E. Vermeulen, Jan N. M. Commandeur, Maarten Honing

Table of contents

	Identification of metabolites	4
	Activity of CYP BM3 mutants towards drugs that are metabolized by human CYPs	7
**Table S1**	Mutants used in this study.	10
**Table S2**	Forward primers used for site-directed mutagenesis.	16
**Table S3**	Compound list including MRM transitions (positive polarity).	18
**Table S4**	Substrate depletion and metabolite formation results for the metabolism of six drugs by the CYP BM3 mutant library.	19
**Table S5**	Metabolic activity and diversity of selected mutants towards dextromethorphan.	24
**Figure S1**	Product profile and conversion of amitriptyline by WT enzyme and all CYP BM3 mutants used in this study.	25
**Figure S2**	Product profile and conversion of buspirone by WT enzyme and all CYP BM3 mutants used in this study.	27
**Figure S3**	Product profile and conversion of dextromethorphan by WT enzyme and all CYP BM3 mutants used in this study.	29
**Figure S4**	Product profile and conversion of diclofenac by WT enzyme and all CYP BM3 mutants used in this study.	31
**Figure S5**	Product profile and conversion of norethisterone by WT enzyme and all CYP BM3 mutants used in this study.	33
**Fig S6**	Mass-trace of *m/z* 294 for all samples.	35

Fig S7 Mass trace of *m/z* 402 for all samples. 36

Fig S8 Mass trace of *m/z* 315 for all samples. 37

References 38

Identification of metabolites

For the confirmation of known and the identification of unknown biotransformation, more detailed MS and MS/MS experiments using the Finnigan LCQ Deca and the Shimadzu IT-TOF were performed. Metabolites were detected for AMI, BUS, CLZ, DEX, DIC, and NET whereas no metabolites were detected for COU.

AMI was metabolized into a total of five products. Three monohydroxylated products, MA1-MA3 (*m/z* 294; $[M+H]^+$), one demethylated product, MA4 (*m/z* 264; $[M+H]^+$), and one di-demethylated product, MA5 (*m/z* 250; $[M+H]^+$), were formed by the different mutants. According to the presence of very intense loss of water (*m/z* 276) and the unique mass fragment at *m/z* 231, for MA1 and MA2 monohydroxylation occurred on the ethylene bridge between the two aromatic rings [1]. Based on HLM reference data from a previous study in our lab [2], (*E*)-10-OH-amitriptyline and (*Z*)-10-OH-amitriptyline were formed. For humans it is known that the (*E*)-10-OH isomer is formed preferentially [3] and it is therefore believed that MA1, which was formed predominantly in the HLM reference incubations, is the (*E*)-10-OH and MA2 is the (*Z*)-10-OH isomer. According to the presence of mass fragments at *m/z* 91, 105, 117, 155, 191, and 233, product MA3 was formed through a methylhydroxylation. The option that this metabolite was formed through a *N*-oxidation was discarded since the thereby expected significant loss of the mass of the oxygen being easily removed from the nitrogen was not observed. In addition, MA3 was also identified in the already mentioned HLM reference incubations which agrees with the findings by Rousu *et al.* [1]. Based upon their masses and the fact that they produced the same mass fragments as the parent molecule, MA4 and MA5 were identified to be the demethylated product nortriptyline and the di-demethylated product, respectively.

BUS was metabolized into a total of six products. Five monohydroxylated products, MB1-MB5 (*m/z* 402; $[M+H]^+$), and one desethyl metabolite, MB6 (*m/z* 360; $[M+H]^+$), were formed by the different mutants. The major mass fragments in the MS/MS spectra of MB1-MB3 were identical (*m/z* 122, 148/150, 219, 238, and 281) and their presence indicates that hydroxylation has not occurred on the 2-(piperazin-1-yl)pyrimidine (PYP) part of the molecule [4]. The presence of very intense loss of water (extra mass fragment at *m/z* 220) in the MS/MS spectra of MB1 and MB2 points to the hydroxylation site being in the cyclopentane part of the azaspirone decane dione (ADD) part of the molecule. The presence of the unique mass fragment at *m/z* 210 in the MS/MS spectrum of MB3 indicates that hydroxylation has also not occurred on the ADD part of the molecule and therefore suggests that hydroxylation might have taken place on the aliphatic chain between the ADD and PYP parts of the molecule. The presence of mass fragments at *m/z* 138, 164/166, 222, and 265 in the MS/MS spectrum of MB4 indicates that hydroxylation occurred on the aromatic ring of the PYP part of the molecule and based upon the results of Zhu *et al.* [4] and in-house HLM reference incubations [2], this metabolite is identified to be 5-hydroxybuspirone (5-OH-BUS). The presence of mass fragments at *m/z* 122, 177, 222, 265, and 277 in the MS/MS spectrum of MB5 indicates that this product is formed through *N*-oxidation of the nitrogen in the piperazine ring which is connected to the aliphatic chain between the ADD and PYP parts of the molecule. MB6 was identified to be the *N*,*N*-desethylbuspirone metabolite [5].

For CLZ, one product with *m/z* value 343.1311 and one product with *m/z* value 313.1207 were found. This corresponds with a previous study in which MT72 was incubated with CLZ and where the metabolites clozapine *N*-oxide (calculated mass (CM) 343.1320: -2.6 ppm) and *N*-desmethylclozapine (CM 313.1215: -2.6 ppm), respectively, were found [6].

DEX was metabolized into a total of six products. Four monohydroxylated products, MD1-MD4 (*m/z* 288; $[M+H]^+$), and two demethylated metabolites, MD5 and MD6 (*m/z* 258; $[M+H]^+$), were formed by the different mutants. The presence of loss of water (extra mass fragment at *m/z* 270) in the MS/MS spectra of MD1-MD3 points to the hydroxylation site being in the aliphatic part of the molecule. However, based on the spectra it is not possible to indicate exactly where the hydroxylation occurred. When comparing the MS/MS spectrum of MD4 with those of the other hydroxylated metabolites unique mass fragments are present at *m/z* value 215 and 271. The presence of these fragments indicates that hydroxylation has occurred on the nitrogen [7]. For MD5 no MS/MS spectrum could be obtained as this metabolite was formed at only very low amounts. HLM reference incubations produced a metabolite with the same mass eluting at the same retention time (data not shown) and based upon these results MD5 was identified as the *O*-demethylated product dextrorphan. The MS/MS spectrum of MD6 displayed mass fragments at *m/z* value 121, 147, 159, 171, 199, 215, 229, and 241. Based upon the HLM reference incubations and MS/MS spectra from literature [8] the metabolite was identified as being the *N*-demethylated product MM.

For DIC during the cocktail screening experiment only a single peak was detected with *m/z* value of 312.0194. This mass indicates that hydroxylation has occurred. In a previous study the metabolism of DIC by different CYP BM3 mutants has already been investigated and it was shown that 4’- and 5-hydroxydiclofenac were formed [9]. In the present study, the peak detected is most likely a combination of these two products. No further efforts have been made to separate both peaks and the combined peaks are therefore simply labeled hydroxydiclofenac (CM 312.0189: -1.6 ppm). NET was metabolized into two monohydroxylated products, MN1 and

MN2 (*m/z* 315; $[M+H]^+$) by the different mutants. Based on previous studies [10, 11] MN1 and MN2 were identified as being 15β- and 16β-hydroxynorethisterone, respectively.

Activity of CYP BM3 mutants towards drugs that are metabolized by human CYPs

The activity of the CYP BM3 mutants towards drugs with a variety of ionic functional groups (acids, bases) that are metabolized by different human CYPs was investigated. The substrates that were selected for the cocktail screening experiment were AMI, BUS, COU, DEX, diclofenac (DIC), and NET. AMI is a positively charged drug for which CYP2C19 appears to be the major determinant of amitriptyline *N*-demethylation at therapeutically relevant drug concentrations [12]. Hydroxylation of AMI is mainly performed by CYP2D6 at therapeutically relevant concentrations [12]. At higher substrate concentrations (> 15 µM) CYP3A4 is also involved in both metabolic pathways. BUS is a positively charged drug that is primarily metabolized by CYP3A4 in human liver microsomes (HLM) [4] into 1-pyrimidinylpiperazine (1-PP), buspirone *N*-oxide (Bu *N*-oxide), 3'-hydroxybuspirone (3'-OH-Bu), 5-hydroxybuspirone (5-OH-Bu), and 6'-hydroxybuspirone (6'-OH-Bu). COU is a neutral drug which in HLM is mainly metabolized by CYP2A6 into 7-hydroxycoumarin (7-OH-COU) whereas CYP1A1, CYP1A2, CYP2B6, and CYP2E1 were shown to form minor amounts of *o*-HPA [13]. COU was also selected as it has been used as a probe to study CYP2A6 activity and CYP2A6-related drug-drug interactions (DDI) in cocktail screens for human CYPs [14–16]. DEX is a positively charged drug which in humans is mainly metabolized by CYP2D6 into the *O*-demethylated product dextrorphan. DEX can also be metabolized by CYP3A4 into the *N*-demethylated product 3-methoxymorphinan (MM) [17]. DEX has been used as a probe to study CYP2D6 activity and CYP2D6-related DDIs

in cocktail screens for human CYPs [18, 19, 16]. DIC was selected to replace CLZ, which is a positively charged drug that is mainly metabolized by CYP3A4 [20], in order to include a negatively charged drug that is metabolized by CYP2C9. DIC is converted in humans into the products 4'-hydroxydiclofenac (4-OH-DIC) by CYP2C9 and 5-hydroxydiclofenac (5-OH-DIC) by CYP3A4 [21]. DIC has been used as a probe to study CYP2C9 activity and CYP2C9-related DDIs in cocktail screens for human CYPs [15, 19]. Experiments were performed to investigate the effect of co-administration of DIC upon the metabolic activity and diversity of MT72-mediated conversion of AMI and BUS which were not found to be affected (data not shown). The neutral drug NET, which is metabolized by CYP3A4 [22], was selected to include a steroid structure in the cocktail mix.

When looking at the activities of the tested CYP BM3 mutants towards the six drugs it appears that the majority of the mutants display activity towards the positively charged drugs AMI, BUS, and DEX. This agrees with the previous finding that M11 predominantly metabolizes positively charged drugs [2] as most of the mutants in the current library are site-directed mutants of M11. Enzymes with a mutation at the 437 position seem to have an increased metabolic efficiency towards the negatively charged drug DIC, which also is in agreement with previous findings [2]. The neutral drug COU is not metabolized by any of the mutants while NET is mainly metabolized by mutants of M01 and M02 and only to a very low extent by mutants of M11 (except for the V87A mutant MT59). It was shown previously that M11 does not metabolize neutral drugs [2].

The results of the screening suggest that the majority of the CYPs screened displays a metabolic profile that is most similar to CYP3A4. BUS, a known CYP3A4 substrate [4], is metabolized by almost all mutants while for DEX the main metabolite formed by the majority of

the mutants is the *N*-demethylated product MM for which it has been reported that formation is catalyzed by CYP3A4 [17]. NET is catalyzed by a selection of the mutants and for this drug it has also been reported that metabolism is catalyzed by CYP3A4 [22]. AMI is metabolized by most of the mutants into the *N*-demethylated product NOR, a reaction which in humans in mainly performed by CYP2C19 [12]. However, it has been reported that at higher AMI concentrations metabolism is mainly performed by CYP3A4 [12]. DIC is metabolized by a selection of the mutant library into the hydroxylated products 4'- and 5-OH-DIC for which in humans formation is catalyzed by CYP2C9 and CYP3A4, respectively [21]. In the present study it was not determined which specific metabolite was formed by the different mutants. A previous study showed that the main metabolite formed for DIC was the 4'-OH-DIC [9] which suggests that the V87F and V87I mutations and mutations at position 437 might make the CYP BM3 mutants more CYP2C9-like. However, formation of the 5-OH-DIC product again implies that the mutants are also 3A4-like.

Table S1 Mutants used in this study

WT	M01	M02	M05	M11	MT21	MT22	MT24	MT28	MT30	MT31	MT32	MT33	MT34	MT35	MT36	MT37	MT38
					Triple L75F	Triple L75W	M11 V87A A264F	M01 T436I	M01 K440S	M01 L437E	M11 L437E	M11 L437N	M11 A74E	M11 L437S	M11 L437T	M11 A74D	M11 S72D
R47	L	L	L	L	L	L	L	L	L	L	L	L	L	L	L	L	L
S53																	
E64				G			G				G	G	G	G	G	G	G
S72																	D
A74													E			D	
L75					F	W											
F81			I	I			I				I	I	I	I	I	I	I
A82																	
L86		I															
F87	V	V	V	V	V	V	A	V	V	V	V	V	V	V	V	V	V
E143				G			G				G	G	G	G	G	G	G
S176																	
L188	Q	Q	Q	Q	Q	Q	Q	Q	Q	Q	Q	Q	Q	Q	Q	Q	Q
Y198				C			C				C	C	C	C	C	C	C
D208																	
T235																	
A264							F										
E267	V		V	V			V	V	V	V	V	V	V	V	V	V	V
H285				Y			Y				Y	Y	Y	Y	Y	Y	Y
N319		T															
A328																	
Q359																	
G415	S		S	S			S	S	S	S	S	S	S	S	S	S	S
T436								I									
L437										E	E	N		S	T		
K440									S								
R471																	
E494																	
A964		V															
S1024																	
G1049			E														
Ref.	[23]	[23]	[23]	[23]						[2]	[2]	[2]	[2]	[2]	[2]	[2]	[2]

Table S1 Mutants used in this study (*continued*)

WT	MT39	MT40	MT41	MT42	MT43	MT44	MT45	MT46	MT47	MT48	MT59	MT61	MT64	MT65	MT66	MT67
	M11<br>V87F<br>A264F	M11<br>A264F	M01<br>A74D	M01<br>A74E	M01<br>S72D	M01<br>S72E	M01<br>L75F	M01<br>L75W	M01<br>V87E	M01<br>L75F<br>V87E	M11<br>V87A	M11<br>V87N	M11<br>V87Q	M11<br>V87E	M11<br>V87G	M11<br>V87H
R47	L	L	L	L	L	L	L	L	L	L	L	L	L	L	L	L
S53																
E64	G	G									G	G	G	G	G	G
S72					D	E										
A74			D	E												
L75							F	W		F						
F81	I	I									I	I	I	I	I	I
A82																
L86																
F87		V	V	V	V	V	V	V	E	E	A	N	Q	E	G	H
E143	G	G									G	G	G	G	G	G
S176																
L188	Q	Q	Q	Q	Q	Q	Q	Q	Q	Q	Q	Q	Q	Q	Q	Q
Y198	C	C									C	C	C	C	C	C
D208																
T235																
A264	F	F														
E267	V	V	V	V	V	V	V	V	V	V	V	V	V	V	V	V
H285	Y	Y									Y	Y	Y	Y	Y	Y
N319																
A328																
Q359																
G415	S	S	S	S	S	S	S	S	S	S	S	S	S	S	S	S
T436																
L437																
K440																
R471																
E494																
A964																
S1024																
G1049																
Ref.			[2]		[2]	[2]					[24]	[24]	[24]	[24]	[24]	[24]

Table S1 Mutants used in this study (*continued*)

WT	MT68	MT69	MT70	MT71	MT72	MT76	MT77	MT78	MT79	MT80	MT81	MT83	MT86	MT87	MT88	MT89
	M11 V87I	M11 V87L	M11 V87K	M11 V87M	M11 V87F	M11 V87W	M11 V87Y	M11 A82W	M11 A82C	M01 A82W	M01 A82C	M11 A264C			M11 V87I L437N	M11 V87I L437S
R47	L	L	L	L	L	L	L	L	L	L	L	L			L	L
S53																
E64	G	G	G	G	G	G	G	G	G			G			G	G
S72																
A74																
L75																
F81	I	I	I	I	I	I	I	I	I			I			I	I
A82								W		W	C					
L86																
F87	I	L	K	M		W	Y	V	V	V	V	V	A	V	I	I
E143	G	G	G	G	G	G	G	G	G			G			G	G
S176																
L188	Q	Q	Q	Q	Q	Q	Q	Q	Q	Q	Q	Q			Q	Q
Y198	C	C	C	C	C	C	C	C	C			C			C	C
D208																
T235																
A264												C				
E267	V	V	V	V	V	V	V	V	V	V	V	V			V	V
H285	Y	Y	Y	Y	Y	Y	Y	Y	Y			Y			Y	Y
N319																
A328													V	V		
Q359																
G415	S	S	S	S	S	S	S	S	S	S	S	S			S	S
T436																
L437															N	S
K440																
R471																
E494																
A964																
S1024																
G1049																
Ref.	[24]	[24]	[24]	[24]	[24]	[24]	[24]	[11]		[11]	[25]				[26]	[26]

Table S1 Mutants used in this study (*continued*)

WT	MT90	MT91	MT92	MT94	MT95	MT96	MT97	MT99	MT100	MT101
	M11<br>V87F L437N	M11<br>V87F L437S	M11<br>A82W L437N	M11<br>A82W V87F	M11<br>A82Y L437S	M11<br>A82Y V87F	M11<br>A82Y V87I	M11<br>S53C Q359C	M11<br>S176C D208C	M11<br>V87A A328I
R47	L	L	L	L	L	L	L	L	L	L
S53								C		
E64	G	G	G	G	G	G	G	G	G	G
S72										
A74										
L75										
F81	I	I	I	I	I	I	I	I	I	I
A82			W	W	Y	Y	Y			
L86										
F87			V		V		I	V	V	A
E143	G	G	G	G	G	G	G	G	G	G
S176									C	
L188	Q	Q	Q	Q	Q	Q	Q	Q	Q	Q
Y198	C	C	C	C	C	C	C	C	C	C
D208									C	
T235										
A264										
E267	V	V	V	V	V	V	V	V	V	V
H285	Y	Y	Y	Y	Y	Y	Y	Y	Y	Y
N319										
A328										I
Q359								C		
G415	S	S	S	S	S	S	S	S	S	S
T436										
L437	N	S	N		S					
K440										
R471										
E494										
A964										
S1024										
G1049										
Ref.	[26]	[26]	[26]		[26]	[27]	[27]			

Table S1 Mutants used in this study (*continued*)

WT	MT102	MT103	MT104	MT105	MT106	MT107	MT108	MT110	MT111	MT112	MT113	MT114
	M02<br>I86L V87A	M02<br>A82W	M01<br>A82W V87A	M01<br>A82W V87I	M01<br>S72I	M01<br>A82W S72I	M11<br>A82W V87A					
R47	L	L	L	L	L	L	L		L	L	L	L
S53												
E64							G					G
S72					I	I						
A74												
L75												
F81							I				I	I
A82		W	W	W		W	W					
L86		I								I		
F87	A	V	A	I	V	V	A	V	V	V	V	V
E143							G					G
S176												
L188	Q	Q	Q	Q	Q	Q	Q		Q	Q	Q	Q
Y198							C					C
D208												
T235								A				
A264												
E267			V	V	V	V	V		V		V	V
H285							Y					Y
N319	T	T								T		
A328								V				
Q359												
G415			S	S	S	S	S		S		S	S
T436												
L437												
K440												
R471								A	A	A	A	A
E494								K	K	K	K	K
A964	V	V								V		
S1024								E	E	E	E	E
G1049											E	
Ref.			[27]	[27]	[27]	[27]	[27]					

Table S1 Mutants used in this study (*continued*)

WT	MT120	MT121	MT122	MT124	MT125	MT126	MT127	MT128	MT129	MT130	MT131	MT132
	M02<br>S72I	M05<br>S72I	M01<br>F81I	M01<br>A82I	M02<br>A82I	M05<br>A82I	M05<br>A82W	M01<br>L86I	M05<br>L86I	M02<br>V87A	M02<br>V87I	M05<br>V87A
R47	L	L	L	L	L	L	L	L	L	L	L	L
S53												
E64												
S72	I	I										
A74												
L75			I									
F81		I				I	I		I			I
A82				I	I	I	W					
L86	I				I			I	I	I	I	
F87	V	V	V	V	V	V	V	V	V	A	I	A
E143												
S176												
L188	Q	Q	Q	Q	Q	Q	Q	Q	Q	Q	Q	Q
Y198												
D208												
T235												
A264												
E267		V	V	V		V	V	V	V			V
H285												
N319	T				T					T	T	
A328												
Q359												
G415		S	S	S		S	S	S	S			S
T436												
L437												
K440												
R471												
E494												
A964	V				V					V	V	
S1024												
G1049		E				E	E		E			E

Table S2 Forward primers used for site-directed mutagenesis

Mutation	Oligonucleotide sequences (5’-3’)	Templates
S53C	CCTGGTTTAGTAACGCGGTACCTATGCAGTCAGCGTCTAATTAAAG	M11
S72I	CGCTTTGATAAAAACTTAATTCAAGCGCTTAAATTT	M02, M05
A74E	GATAAAAACTTAAGTCAAGAACTTAAATTGTACGT	M01
L75F	AACTTAAGTCAAGCGTTTAAATTTGTACGTGAT	Triple mutant (R47L, F87V, L188Q), M01
L75W	AACTTAAGTCAAGCTTGGAAATTTGTACGTGAT	Triple mutant (R47L, F87V, L188Q), M01
F81I	GTACGTGATATTGCCGGCGACGGGTTAGTAAC	M01
F81I	GTACGTGATATTGCCGGCGACGGGATAGTAAC	M02
A82I	GTCAAGCGCTTAAATTTGTTCGCGATTTTATAGGAGACGGG	M01, M02
A82I	GTCAAGCGCTTAAATTTGTTCGCGATATTATAGGAGACGGG	M05
A82C	GTCAAGCGCTTAAATTTGTTCGCGATTATTGCGGAGACGGG	M11
A82W	GTCAAGCGCTTAAATTTGTTCGCGATTTTTGGGGAGACGGG	M02
A82W	GTCAAGCGCTTAAATTTGTTCGCGATTATTGGGGAGACGGG	M05, MT59 (M11 V87F)
I86L V87A	GCAGGAGACGGGTTAGCCACTAGTTGGACGCAT	M02
L86I	GCAGGAGACGGGATAGTGACGTCCTGGACGCATG	M01, M05
F87A/V87A	GCAGGAGACGGGTTAGCCACTAGTTGGACGCAT	WT, M05
F87V	GCAGGAGACGGGTTAGTAACTAGTTGGACGCAT	WT

V87A	GCAGGAGACGGGATAGCCACTAGTTGGACGCAT	M02
V87E	GCAGGAGACGGGTTAGAGACTAGTTGGACGCAT	M01
V87I	GCAGGAGACGGGATAATCACTAGTTGGACGCAT	M02
S176C	GCCTCATCCATTTATCACGTGTATGGTCCGTGCACTGG	M11
D208C	CAAGCGCCAGTTTCAAGAATGCATCAAGGTGATGAACGACC	M11
T235A	GCGATGATTTGCTAGCGCATATGCTAAACGGA	WT
A264C	ATTACATTCTTAATTTGTGGCCACGTAACAACA	M11
A264F	ATTACATTCTTAATTTTTGGCCACGTAACAACA	M11, MT59 (M11 V87A), MT72 (M11 V87F)
A328I	CGCTTATGGCCAACTATTCCTGCGTTTTCCC	MT59 (M11 V87A)
A328V	CGCTTATGGCCGACTGTTCCTGCGTTTTCCC	WT
Q359C	CGAACTAATGGTTCTGATTCCTTGTCTACACCGTGATAAAACAATTTGG	M11
T436I	CTCGATATTAAAGAGATCTTAACGTTAAAACCT	M01
K440S	GAAACTTTAACGTTAAGCCCTGAAGGCTTTGTG	M01
R471A	CAGTCTGCTAAAAAAGTCGCGAAAAAGGCA	M11
E494K	GGTTCAAATATGGGAACAGCCAAGGGAACGGCGCGT	M11
S1024E	GCTGACGTCCACCAAGTGGAGGAAGCAGACGCTCGC	M11

Table S3 Compound list including MRM transitions (positive polarity)

Compound	Q1	Q3	Declustering Potential	Collision Energy	Exit Collision Potential
Amitriptyline (AMI)	278.3	115.2	36	61	6
Nortriptyline (NOR)	264.2	117.1	86	29	10
Buspirone (BUS)	386.3	148.1	140	47	8
5-Hydroxybuspirone (5-OH-BUS)	402.3	138.1	136	45	10
Coumarine (COU)	147.0	91.1	66	33	6
7-Hydroxycoumarine (7-OH-COU)	163.0	107.2	91	31	6
Dextromethorphan (DEX)	272.2	171.3	131	53	10
Methoxymorphinan (MM)	258.2	215.3	96	31	10
Diclofenac (DIC)	296.1	215.2	56	27	12
4-Hydroxydiclofenac (4-OH-DIC)	312.0	231.0	66	45	12
Minaprine (MIN)	299.3	77.0	71	77	6
Norethisterone (NET)	299.3	109.0	91	37	8
16β-Hydroxynorethisterone (16β-OH-NET)	297.2	109.1	121	35	8

Table S4 Substrate depletion and metabolite formation results for the metabolism of six drugs by the CYP BM3 mutant library

Mutant	ΔAMI (μM)	NOR (μM)	ΔBUS (μM)	5-OH-BUS (μM)	ΔCOU (μM)	7-OH-COU (μM)	ΔDEX (μM)	MM (μM)	ΔDIC (μM)	OH-DIC (μM)	ΔNET (μM)	16β-OH-NET (μM)
WT	3.5 ± 5.7	0.2 ± 0.1	6.3 ± 2.8	0.1 ± 0.1	21.2 ± 9.3	NPD	-18.4 ± 6.7	0.1 ± 0.1	9.3 ± 10.7	0.1 ± 0.1	25.7 ± 13.2	NPD
M01	57.3 ± 7.5	36.2 ± 5.2	11.8 ± 7.7	4.7 ± 0.6	-12.9 ± 12.4	NPD	6.9 ± 8.1	13.7 ± 1.0	-6.8 ± 7.6	1.3 ± 0.3	38.6 ± 8.4	NPD
M02	57.2 ± 7.5	44.1 ± 1.2	16.4 ± 7.0	4.8 ± 0.2	17.6 ± 25.5	NPD	3.2 ± 8.4	17.2 ± 0.7	7.1 ± 6.8	1.0 ± 0.1	56.6 ± 5.9	NPD
M05	76.3 ± 8.1	45.6 ± 8.1	28.6 ± 7.9	8.8 ± 0.6	27.1 ± 15.5	NPD	4.3 ± 5.5	29.0 ± 1.7	15.4 ± 14.9	1.1 ± 0.2	31.0 ± 17.6	NPD
M11	79.2 ± 9.3	49.1 ± 0.8	29.6 ± 5.7	10.0 ± 0.4	19.5 ± 23.8	NPD	6.2 ± 3.7	29.8 ± 1.3	13.0 ± 10.2	2.4 ± 0.2	29.0 ± 9.4	NPD
MT21	6.9 ± 7.7	1.1 ± 0.1	11.9 ± 5.1	0.1 ± 0.1	5.2 ± 13.9	NPD	-10.6 ± 3.0	0.2 ± 0.1	7.3 ± 2.2	0.1 ± 0.1	21.1 ± 10.9	NPD
MT22	3.0 ± 8.3	0.4 ± 0.1	6.4 ± 8.3	0.1 ± 0.1	0.8 ± 15.7	NPD	-11.4 ± 4.2	0.2 ± 0.1	6.2 ± 8.2	0.1 ± 0.1	35.7 ± 12.0	NPD
MT24	6.3 ± 11.0	0.9 ± 0.1	15.2 ± 7.1	0.1 ± 0.1	27.5 ± 22.7	NPD	-20.7 ± 5.4	0.2 ± 0.1	13.8 ± 11.8	0.1 ± 0.1	18.4 ± 12.3	NPD
MT28	30.2 ± 2.7	18.0 ± 0.7	21.2 ± 7.4	7.5 ± 0.8	1.7 ± 25.9	NPD	-4.4 ± 4.6	5.6 ± 0.3	1.3 ± 11.3	1.0 ± 0.1	17.4 ± 9.5	NPD
MT30	41.9 ± 5.7	32.0 ± 3.0	7.6 ± 7.4	2.4 ± 0.1	7.6 ± 19.0	NPD	2.8 ± 4.1	9.4 ± 1.0	-0.1 ± 4.7	0.5 ± 0.1	-1.1 ± 9.9	NPD
MT31	42.0 ± 7.5	31.4 ± 2.7	5.2 ± 6.8	1.7 ± 0.1	-1.8 ± 16.0	NPD	7.9 ± 9.9	8.9 ± 0.6	-3.0 ± 7.0	0.7 ± 0.2	-10.8 ± 14.4	NPD
MT32	68.3 ± 6.4	41.6 ± 0.9	-2.6 ± 8.0	2.0 ± 0.1	-3.5 ± 11.9	NPD	21.4 ± 6.1	20.6 ± 0.7	9.9 ± 4.4	9.4 ± 0.5	19.3 ± 6.9	NPD
MT33	67.2 ± 3.9	39.7 ± 1.3	10.8 ± 3.8	2.1 ± 0.1	7.6 ± 14.2	NPD	15.0 ± 5.6	17.8 ± 1.2	11.2 ± 4.0	7.9 ± 0.1	7.0 ± 7.9	NPD
MT34	61.1 ± 5.6	41.2 ± 1.1	5.2 ± 4.6	1.4 ± 0.1	-19.6 ± 6.8	NPD	9.2 ± 5.1	14.2 ± 0.6	3.3 ± 4.5	2.8 ± 0.2	8.7 ± 8.6	NPD
MT35	77.7 ± 4.5	46.3 ± 2.2	15.3 ± 3.8	5.8 ± 0.4	3.2 ± 11.2	NPD	20.4 ± 5.8	24.2 ± 1.0	4.3 ± 4.7	4.5 ± 0.3	4.4 ± 14.2	NPD

MT36	62.3 ± 8.4	38.2 ± 6.1	6.9 ± 8.0	1.2 ± 0.1	-4.7 ± 14.9	NPD	13.3 ± 9.7	12.6 ± 1.4	-0.1 ± 4.8	1.9 ± 0.1	16.3 ± 13.3	NPD
MT37	70.2 ± 5.8	39.3 ± 3.7	8.6 ± 7.5	1.9 ± 0.1	-3.4 ± 13.5	NPD	10.4 ± 8.9	16.1 ± 2.7	3.0 ± 8.6	2.9 ± 0.1	16.3 ± 13.3	NPD
MT38	55.1 ± 5.8	40.4 ± 0.8	6.3 ± 3.9	0.3 ± 0.1	-10.0 ± 20.1	NPD	12.7 ± 9.5	16.7 ± 2.1	0.5 ± 5.5	0.3 ± 0.1	6.9 ± 9.6	NPD
MT39	1.9 ± 7.4	0.6 ± 0.1	9.4 ± 0.8	0.1 ± 0.1	5.4 ± 13.2	NPD	3.6 ± 6.5	0.3 ± 0.1	2.3 ± 6.9	0.1 ± 0.1	0.5 ± 15.2	NPD
MT40	5.4 ± 4.2	0.5 ± 0.1	1.3 ± 3.2	0.1 ± 0.1	8.4 ± 11.8	NPD	-3.7 ± 8.8	0.3 ± 0.1	1.2 ± 8.6	0.1 ± 0.1	1.8 ± 6.3	NPD
MT41	46.8 ± 11.0	30.7 ± 6.4	13.1 ± 16.5	3.0 ± 0.9	-3.1 ± 9.3	NPD	10.0 ± 7.5	8.5 ± 0.8	-1.6 ± 3.3	0.6 ± 0.1	2.7 ± 10.4	NPD
MT42	68.7 ± 17.0	51.9 ± 4.4	4.4 ± 9.7	0.6 ± 0.1	-2.9 ± 12.0	NPD	18.2 ± 10.0	16.4 ± 0.5	-1.7 ± 10.0	0.7 ± 0.1	-2.9 ± 12.2	NPD
MT43	32.2 ± 3.9	20.3 ± 0.9	14.2 ± 2.1	0.2 ± 0.1	3.2 ± 10.1	NPD	4.3 ± 7.8	6.1 ± 0.3	-5.8 ± 6.8	0.1 ± 0.1	-5.0 ± 6.2	NPD
MT44	44.0 ± 7.6	30.0 ± 1.5	7.2 ± 10.3	2.3 ± 0.1	7.5 ± 9.9	NPD	15.8 ± 7.7	7.9 ± 0.7	-2.9 ± 9.2	0.5 ± 0.1	6.1 ± 16.7	NPD
MT45	3.6 ± 7.2	8.3 ± 0.3	7.4 ± 4.7	0.1 ± 0.1	3.0 ± 15.2	NPD	2.0 ± 8.8	0.9 ± 0.1	-5.4 ± 6.3	0.5 ± 0.1	-11.0 ± 12.1	NPD
MT46	34.4 ± 10.4	24.1 ± 3.5	13.2 ± 3.9	3.3 ± 0.3	-9.9 ± 20.9	NPD	4.0 ± 4.4	1.9 ± 0.2	-3.3 ± 5.2	0.5 ± 0.1	-5.2 ± 3.9	NPD
MT47	33.0 ± 2.9	32.7 ± 2.9	6.7 ± 6.4	5.4 ± 0.3	14.3 ± 16.1	NPD	0.4 ± 7.0	2.3 ± 0.3	4.0 ± 5.5	0.1 ± 0.1	-1.2 ± 9.9	NPD
MT48	57.5 ± 3.3	31.6 ± 2.7	1.2 ± 4.0	0.3 ± 0.1	5.1 ± 14.5	NPD	0.1 ± 8.9	2.0 ± 0.3	-7.4 ± 7.8	0.2 ± 0.2	0.2 ± 10.12	NPD
MT59	49.8 ± 7.8	42.1 ± 3.5	22.5 ± 3.6	3.1 ± 0.3	-0.8 ± 16.2	NPD	38.4 ± 13.9	49.6 ± 2.5	0.9 ± 5.9	1.1 ± 0.1	35.5 ± 4.5	NPD
MT61	25.4 ± 6.9	28.1 ± 9.9	7.9 ± 8.3	1.1 ± 0.4	4.6 ± 15.9	NPD	5.9 ± 4.9	10.0 ± 3.0	-3.3 ± 7.2	0.5 ± 0.3	2.1 ± 8.6	NPD
MT64	8.4 ± 2.5	7.7 ± 0.7	6.1 ± 5.9	0.6 ± 0.1	4.6 ± 9.9	NPD	4.5 ± 3.1	3.3 ± 0.1	1.5 ± 7.9	1.0 ± 0.1	0.6 ± 6.2	NPD
MT65	31.5 ± 7.5	27.1 ± 2.9	3.2 ± 5.4	0.2 ± 0.1	-0.9 ± 17.5	NPD	10.6 ± 6.4	12.6 ± 1.0	-4.7 ± 8.6	0.1 ± 0.1	-6.3 ± 3.6	NPD
MT66	25.2 ± 8.5	14.2 ± 1.1	25.3 ± 4.0	0.4 ± 0.1	0.5 ± 20.3	NPD	50.6 ± 10.3	31.9 ± 1.2	2.9 ± 8.8	0.1 ± 0.2	13.5 ± 11.6	NPD

MT67	22.9 ± 9.7	19.4 ± 1.3	36.6 ± 5.6	10.2 ± 1.3	10.9 ± 14.6	NPD	4.3 ± 6.2	4.0 ± 0.3	13.1 ± 4.3	35.5 ± 1.0	-3.7 ± 9.5	NPD
MT68	64.9 ± 2.3	50.9 ± 3.1	17.5 ± 10.7	10.0 ± 1.1	1.6 ± 8.1	NPD	23.6 ± 4.8	32.4 ± 2.3	3.7 ± 12.5	5.0 ± 0.6	6.0 ± 21.2	NPD
MT69	3.7 ± 6.8	16.7 ± 0.7	14.6 ± 2.0	9.6 ± 0.9	12.4 ± 11.5	NPD	-6.9 ± 4.4	1.9 ± 0.3	10.3 ± 1.5	5.5 ± 0.3	9.1 ± 3.4	NPD
MT70	13.9 ± 14.0	6.4 ± 1.3	3.6 ± 9.3	2.9 ± 0.1	5.4 ± 6.1	NPD	-0.2 ± 7.5	0.7 ± 0.1	-1.2 ± 4.0	0.1 ± 0.1	-1.3 ± 7.0	NPD
MT71	29.4 ± 14.8	4.7 ± 1.0	4.7 ± 0.1	0.7 ± 0.1	-3.4 ± 5.8	NPD	12.0 ± 5.9	2.2 ± 0.2	5.6 ± 2.9	1.0 ± 0.2	1.6 ± 3.6	NPD
MT72	33.4 ± 14.9	23.7 ± 2.5	52.3 ± 4.4	14.5 ± 0.7	18.9 ± 10.1	NPD	4.7 ± 10.6	5.3 ± 0.1	19.6 ± 2.7	38.7 ± 0.7	8.2 ± 4.5	NPD
MT76	-2.7 ± 4.3	1.0 ± 0.3	2.3 ± 4.8	0.1 ± 0.1	7.2 ± 11.1	NPD	2.5 ± 10.4	0.7 ± 0.1	-3.2 ± 6.5	0.1 ± 0.1	-6.9 ± 4.6	NPD
MT77	27.5 ± 7.1	28.3 ± 9.5	9.9 ± 10.6	4.9 ± 0.8	4.2 ± 7.9	NPD	3.3 ± 17.5	15.8 ± 2.9	7.1 ± 7.4	2.1 ± 0.5	11.3 ± 15.8	NPD
MT78	55.4 ± 8.7	48.2 ± 6.4	12.6 ± 3.5	0.9 ± 0.1	18.6 ± 8.5	NPD	17.3 ± 4.5	25.5 ± 1.8	5.9 ± 6.4	0.8 ± 0.2	16.5 ± 6.6	NPD
MT79	74.1 ± 9.8	37.1 ± 15.1	11.4 ± 3.0	9.9 ± 0.7	-2.2 ± 12.4	NPD	20.3 ± 8.5	20.5 ± 3.4	-5.2 ± 2.5	2.4 ± 0.3	-2.6 ± 3.0	NPD
MT80	42.6 ± 9.1	55.1 ± 1.5	2.6 ± 9.2	1.0 ± 0.0	-10.6 ± 10.9	NPD	22.7 ± 7.7	23.8 ± 1.7	-5.7 ± 10.8	0.1 ± 0.1	1.8 ± 14.0	NPD
MT81	52.5 ± 4.7	31.7 ± 2.0	9.8 ± 2.9	2.1 ± 0.1	10.2 ± 9.6	NPD	15.7 ± 7.0	13.7 ± 1.8	5.5 ± 7.1	1.6 ± 0.2	6.5 ± 6.2	NPD
MT83	13.6 ± 14.8	0.5 ± 0.1	4.6 ± 9.4	0.1 ± 0.1	6.0 ± 5.3	NPD	3.0 ± 9.0	0.3 ± 0.1	6.3 ± 5.1	0.1 ± 0.1	1.0 ± 7.5	NPD
MT86	6.2 ± 9.7	0.4 ± 0.1	14.0 ± 26.6	0.5 ± 0.2	-3.1 ± 2.9	NPD	-19.5 ± 8.8	0.2 ± 0.1	8.9 ± 9.0	0.1 ± 0.1	0.1 ± 5.7	NPD
MT87	5.4 ± 8.2	0.4 ± 0.1	23.9 ± 3.0	0.1 ± 0.1	3.3 ± 12.5	NPD	0.5 ± 3.1	0.2 ± 0.1	0.8 ± 4.5	0.1 ± 0.1	5.2 ± 6.4	NPD
MT88	77.5 ± 11.6	35.1 ± 11.2	7.6 ± 7.8	1.2 ± 0.1	11.1 ± 20.3	NPD	28.1 ± 11.9	35.4 ± 3.1	20.5 ± 9.3	21.3 ± 1.9	15.3 ± 12.1	NPD
MT89	70.1 ± 5.8	34.5 ± 9.5	0.8 ± 7.5	3.7 ± 0.2	-17.9 ± 13.9	NPD	40.0 ± 13.0	41.0 ± 3.3	-1.7 ± 9.1	8.6 ± 0.4	-12.3 ± 18.5	NPD
MT90	17.8 ± 3.0	7.4 ± 1.6	13.7 ± 7.8	6.8 ± 1.3	1.9 ± 11.7	NPD	0.6 ± 9.7	2.3 ± 0.6	10.9 ± 7.3	27.6 ± 5.3	2.5 ± 7.0	NPD

MT91	20.4 ± 19.4	18.5 ± 0.1	37.3 ± 20.4	22.2 ± 1.2	0.5 ± 6.6	NPD	3.3 ± 14.7	4.3 ± 0.1	10.2 ± 3.3	17.1 ± 1.1	1.7 ± 12.5	NPD
MT92	23.6 ± 15.9	20.3 ± 5.0	4.4 ± 13.7	0.2 ± 0.1	5.5 ± 17.8	NPD	-0.2 ± 9.8	5.1 ± 1.0	8.6 ± 14.1	1.1 ± 0.2	12.0 ± 23.0	NPD
MT94	27.6 ± 5.4	18.1 ± 2.8	39.5 ± 6.8	0.7 ± 0.1	-15.4 ± 16.9	NPD	4.6 ± 11.5	5.1 ± 0.9	7.2 ± 4.7	7.8 ± 1.2	10.5 ± 7.5	NPD
MT95	53.6 ± 22.0	54.3 ± 6.1	9.0 ± 8.5	0.4 ± 0.1	9.4 ± 2.5	NPD	-0.8 ± 8.2	3.9 ± 0.6	4.3 ± 7.4	0.1 ± 0.1	7.4 ± 9.9	NPD
MT96	43.1 ± 7.0	7.1 ± 2.1	4.4 ± 13.8	0.5 ± 0.1	-4.6 ± 19.8	NPD	17.4 ± 13.4	2.1 ± 0.2	6.1 ± 7.4	0.8 ± 0.1	-9.4 ± 22.7	NPD
MT97	21.3 ± 9.7	29.9 ± 0.6	0.4 ± 7.7	0.9 ± 0.1	-5.1 ± 7.6	NPD	3.9 ± 8.2	5.6 ± 0.6	1.0 ± 4.7	0.5 ± 0.1	-0.1 ± 4.7	NPD
MT99	68.9 ± 8.9	49.7 ± 6.8	16.7 ± 10.6	8.5 ± 0.4	4.0 ± 24.2	NPD	21.5 ± 12.4	14.5 ± 2.9	-0.4 ± 13.5	2.2 ± 0.5	3.9 ± 4.3	NPD
MT100	32.4 ± 21.0	42.1 ± 1.7	14.4 ± 8.7	6.5 ± 0.1	-1.3 ± 12.4	NPD	-4.8 ± 8.9	15.1 ± 1.3	3.8 ± 8.1	1.5 ± 0.5	11.6 ± 13.4	NPD
MT101	0.6 ± 11.3	4.2 ± 0.5	3.8 ± 23.3	1.6 ± 0.3	-5.7 ± 13.9	NPD	7.0 ± 14.3	2.4 ± 0.1	-8.6 ± 11.2	1.8 ± 0.2	5.6 ± 4.4	NPD
MT102	2.1 ± 6.1	1.5 ± 0.1	3.1 ± 5.5	0.2 ± 0.1	-12.1 ± 14.2	NPD	-2.2 ± 6.3	0.9 ± 0.1	6.2 ± 7.0	0.1 ± 0.1	81.6 ± 10.1	NPD
MT103	40.6 ± 13.2	33.9 ± 5.5	7.0 ± 5.6	0.6 ± 0.1	-12.6 ± 19.9	NPD	8.2 ± 8.5	5.9 ± 0.7	-2.1 ± 4.4	0.1 ± 0.1	47.8 ± 8.2	NPD
MT104	67.6 ± 5.0	60.7 ± 2.6	14.5 ± 1.7	0.7 ± 0.1	15.7 ± 12.0	NPD	27.8 ± 2.7	34.4 ± 0.3	10.2 ± 2.2	0.1 ± 0.1	49.4 ± 4.3	NPD
MT105	71.5 ± 2.0	65.5 ± 5.7	14.2 ± 3.8	7.3 ± 0.6	-6.2 ± 6.3	NPD	46.6 ± 4.4	46.8 ± 2.7	-4.7 ± 7.2	1.8 ± 0.1	-4.0 ± 7.1	NPD
MT106	37.7 ± 18.5	30.5 ± 2.2	9.3 ± 0.2	2.1 ± 0.2	11.9 ± 12.2	NPD	0.9 ± 6.7	5.3 ± 0.3	4.3 ± 5.6	3.6 ± 0.2	2.1 ± 6.5	NPD
MT107	77.7 ± 4.4	69.3 ± 2.5	5.3 ± 2.0	0.7 ± 0.1	17.4 ± 7.1	NPD	13.0 ± 3.8	19.0 ± 1.4	-0.2 ± 3.8	0.4 ± 0.1	25.4 ± 5.2	NPD
MT108	46.5 ± 15.8	35.7 ± 4.3	41.5 ± 11.3	0.3 ± 0.1	-5.6 ± 15.4	NPD	17.0 ± 6.6	19.6 ± 0.9	-1.4 ± 4.8	0.1 ± 0.1	10.0 ± 6.0	NPD
MT110	1.4 ± 3.0	0.7 ± 0.1	2.6 ± 5.1	0.5 ± 0.1	-6.9 ± 22.6	NPD	2.0 ± 4.2	0.6 ± 0.1	-1.1 ± 6.9	0.1 ± 0.1	4.5 ± 5.3	NPD
MT111	19.7 ± 4.9	16.7 ± 0.7	6.6 ± 4.1	1.7 ± 0.1	6.2 ± 14.4	NPD	5.7 ± 4.2	5.2 ± 0.3	-2.4 ± 2.7	0.5 ± 0.1	-2.4 ± 3.7	NPD

MT112	33.8 ± 17.9	13.1 ± 4.4	58.8 ± 3.7	1.3 ± 0.4	13.3 ± 13.6	NPD	4.9 ± 6.1	5.0 ± 0.8	-3.0 ± 6.4	0.5 ± 0.2	-0.1 ± 8.3	NPD
MT113	27.9 ± 2.4	25.7 ± 1.0	5.9 ± 3.1	2.1 ± 0.1	-5.2 ± 15.1	NPD	6.0 ± 5.3	9.9 ± 0.8	3.2 ± 3.4	0.4 ± 0.1	2.9 ± 1.3	NPD
MT114	46.7 ± 24.1	33.5 ± 7.3	66.6 ± 4.1	2.4 ± 1.2	8.6 ± 23.6	NPD	14.2 ± 11.2	17.2 ± 2.3	-6.0 ± 7.4	1.0 ± 0.1	-7.2 ± 9.1	NPD
MT120	27.1 ± 7.6	29.7 ± 0.7	7.5 ± 6.4	0.3 ± 0.1	-4.7 ± 17.2	NPD	-2.5 ± 5.3	4.1 ± 0.1	2.4 ± 7.9	1.3 ± 0.3	12.8 ± 4.5	NPD
MT121	66.1 ± 11.9	55.5 ± 4.7	14.1 ± 18.8	1.7 ± 0.2	-14.0 ± 7.7	NPD	-0.7 ± 5.9	3.1 ± 0.5	-2.7 ± 2.8	2.5 ± 0.1	-7.8 ± 8.2	NPD
MT122	53.8 ± 14.5	39.2 ± 5.7	7.9 ± 5.5	4.1 ± 0.3	-11.5 ± 9.6	NPD	17.3 ± 5.6	21.0 ± 0.8	-0.6 ± 4.0	0.8 ± 0.2	-7.3 ± 9.1	NPD
MT124	41.3 ± 4.5	39.6 ± 2.5	1.1 ± 0.9	0.5 ± 0.1	-4.3 ± 22.8	NPD	6.4 ± 4.8	8.6 ± 1.0	-3.3 ± 3.7	0.9 ± 0.1	-0.8 ± 3.5	NPD
MT125	42.0 ± 13.1	40.7 ± 3.0	-1.7 ± 3.3	0.6 ± 0.1	-1.3 ± 24.9	NPD	-1.6 ± 6.6	6.8 ± 0.7	-8.4 ± 4.9	0.3 ± 0.1	83.4 ± 2.5	NPD
MT126	81.7 ± 6.0	77.6 ± 1.8	6.2 ± 5.0	0.7 ± 0.1	2.9 ± 17.2	NPD	19.4 ± 6.5	28.7 ± 1.6	-0.8 ± 4.9	0.2 ± 0.1	3.2 ± 6.3	NPD
MT127	73.5 ± 2.8	59.5 ± 6.0	14.7 ± 3.3	1.9 ± 0.4	9.6 ± 7.2	NPD	30.1 ± 6.0	34.8 ± 2.0	11.2 ± 2.8	3.1 ± 0.6	15.7 ± 7.7	NPD
MT128	12.1 ± 6.2	8.9 ± 0.5	-0.6 ± 4.0	0.4 ± 0.1	0.7 ± 16.3	NPD	7.0 ± 11.6	4.7 ± 0.1	-4.9 ± 9.7	0.1 ± 0.1	-2.8 ± 10.8	NPD
MT129	64.2 ± 24.8	29.4 ± 9.0	56.3 ± 9.3	0.9 ± 0.3	-14.9 ± 24.2	NPD	37.9 ± 7.5	38.6 ± 2.4	0.2 ± 8.0	0.6 ± 0.1	3.3 ± 14.6	NPD
MT130	3.7 ± 3.4	5.9 ± 0.6	-1.0 ± 6.7	0.2 ± 0.1	-30.0 ± 17.4	NPD	8.5 ± 6.5	3.5 ± 0.3	-6.6 ± 6.4	0.1 ± 0.1	-3.3 ± 6.0	NPD
MT131	31.2 ± 16.8	20.5 ± 3.0	10.4 ± 4.1	2.2 ± 0.7	-2.9 ± 19.8	NPD	-1.3 ± 8.1	4.0 ± 0.2	4.1 ± 3.4	0.5 ± 0.1	15.8 ± 4.9	NPD
MT132	10.9 ± 19.5	11.5 ± 2.9	5.1 ± 4.4	0.6 ± 0.1	1.7 ± 5.0	NPD	17.5 ± 4.3	14.8 ± 1.5	-0.1 ± 5.3	0.1 ± 0.1	7.3 ± 8.5	NPD
Averaged STD	9.1	3.0	6.9	0.3	14.0	NA	7.5	1.0	6.6	0.4	8.9	NA

NPD: No peak detected   NA: Not applicable

STD: Standard deviation

Table S5 Metabolic activity and diversity of selected mutants towards dextromethorphan

DEX Mutant	UHPLC (%) [a]	UHPLC MM (µM) [b]	IT-TOF (%) [c]	MD1 %	MD2 %	MD3 %	MD4 %	MD5 %	MD6 %
MT66	**50.6**	31.9	**67.9**	11	14	21	19	-	35
MT105	**46.6**	46.7	**53.7**	-	-	-	-	-	100
MT89	**40.0**	41.0	**46.7**	3	-	-	-	-	97
MT59	**38.4**	49.6	**51.8**	2	-	1	8	-	89
MT129	**37.9**	38.6	**43.0**	1	-	-	2	-	97
MT127	**30.1**	34.8	**43.3**	6	1	-	2	-	91
MT88	**28.1**	35.4	**38.2**	2	-	-	-	1	97
MT104	**27.8**	34.4	**41.8**	-	-	-	6	-	94

[a] The substrate depletion is calculated by using the average peak area of the parent at 60 min and at time zero. Values are expressed in percentages of the average peak area of the parent at time zero. Measurements were performed in triplicate and results were obtained during analysis of the cocktail incubations by UHPLC-MS/MS

[b] The amount of product formed is calculated by using the average peak area of the metabolite at 60 min. Measurements were performed in triplicate and results were obtained during analysis of the cocktail incubations by UHPLC-MS/MS

[c] The substrate depletion is calculated by using the peak area from the MS signal of the parent at 60 min and the sum of the peak areas from the MS signals of the parent and its metabolites at 60 min. The substrate depletion is expressed as percentage of parent that has been converted into metabolites. Results were obtained during analysis of the cocktail incubation by LC-MS/MS on the IT-TOF.

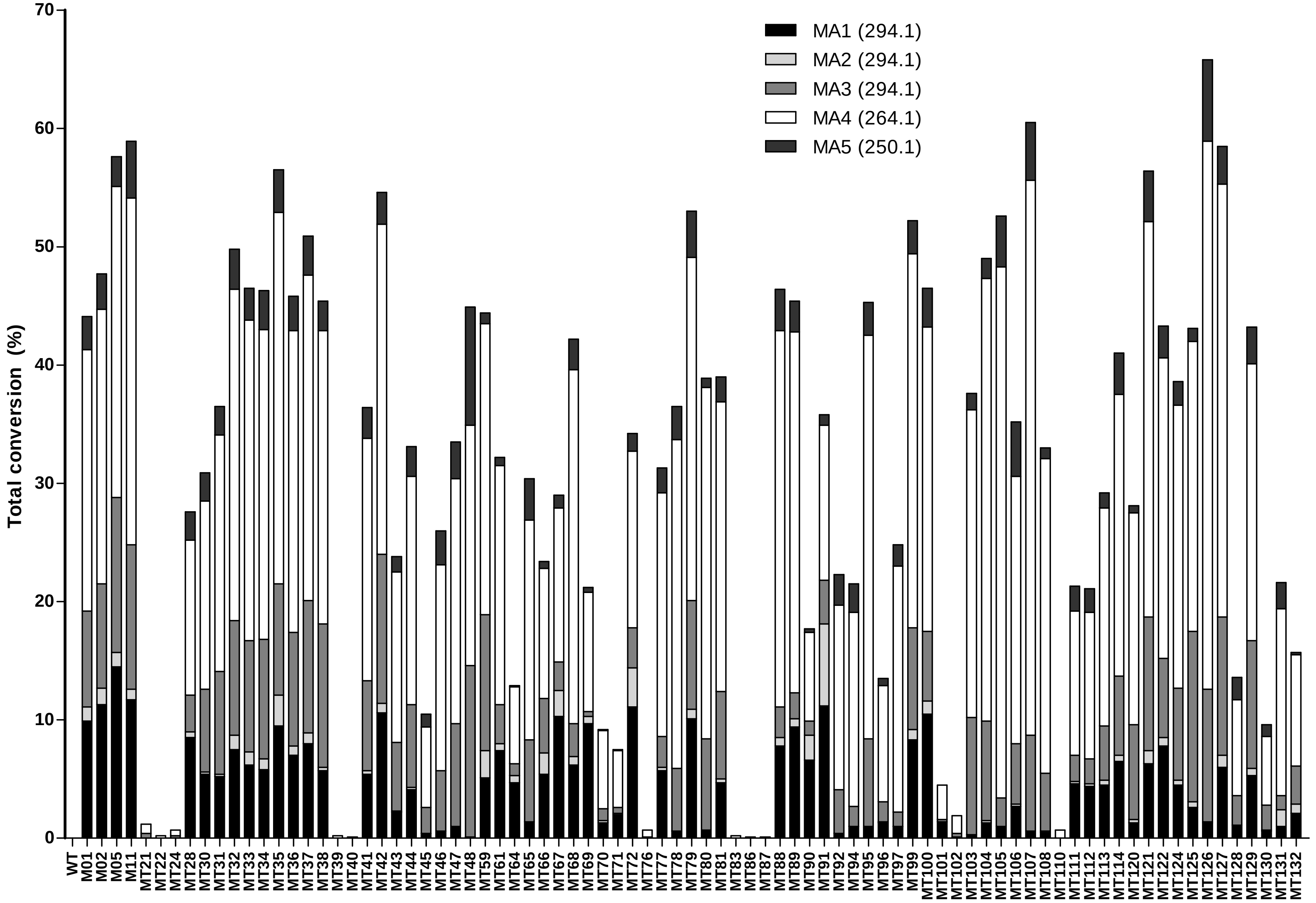

Fig. S1 Product profile and conversion of amitriptyline by WT enzyme and all CYP BM3 mutants used in this study. Conversions

were determined by integrating MS signals of the parent peak and all metabolites in the corresponding extracted ion chromatograms and are expressed as percentage of the parent metabolized after 60 min. Product selectivity in % was calculated using the MS signals of all metabolites

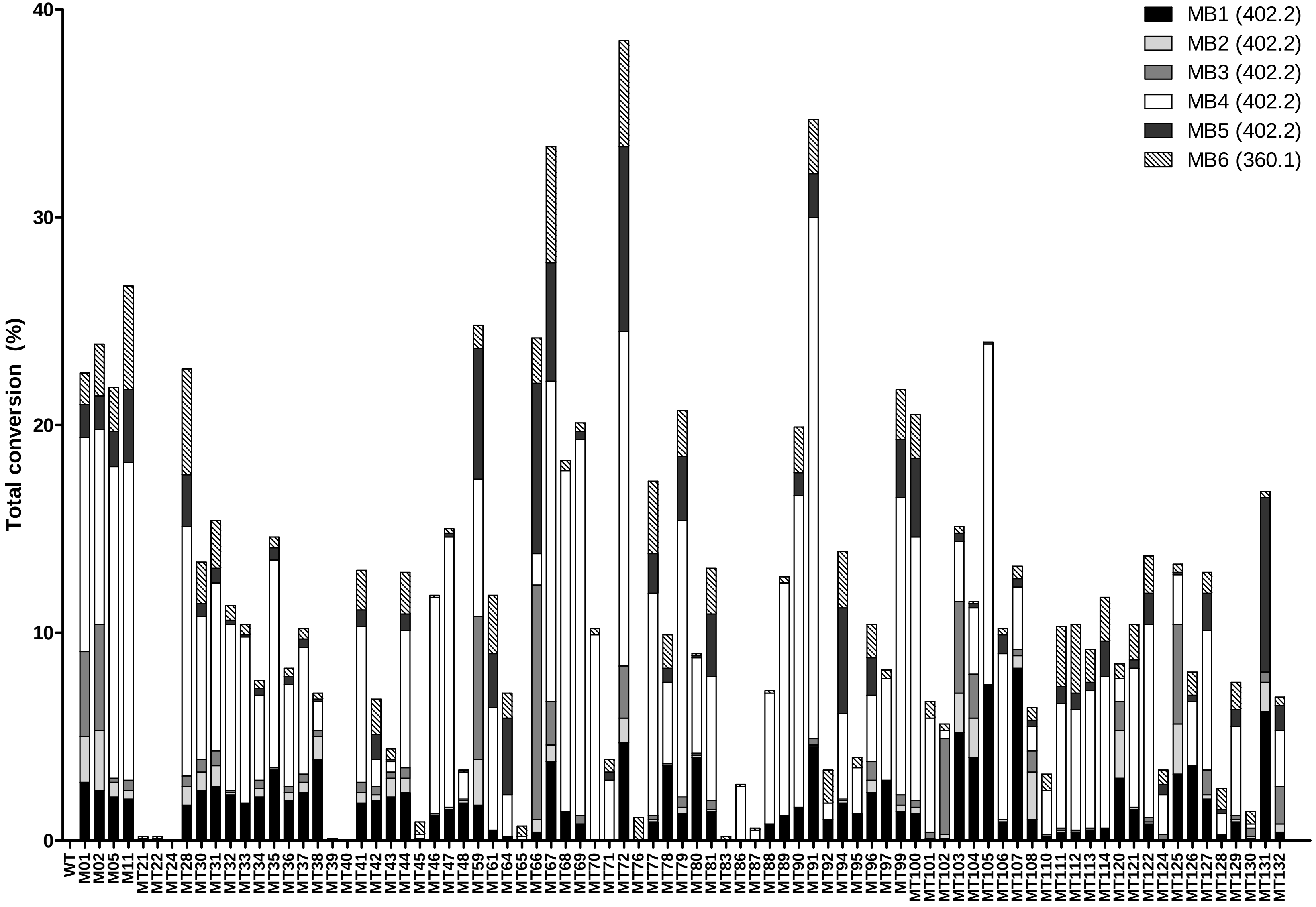

Fig. S2 Product profile and conversion of buspirone by WT enzyme and all CYP BM3 mutants used in this study. Conversions were

determined by integrating MS signals of the parent peak and all metabolites in the corresponding extracted ion chromatograms and are expressed as percentage of the parent metabolized after 60 min. Product selectivity in % was calculated using the MS signals of all metabolites

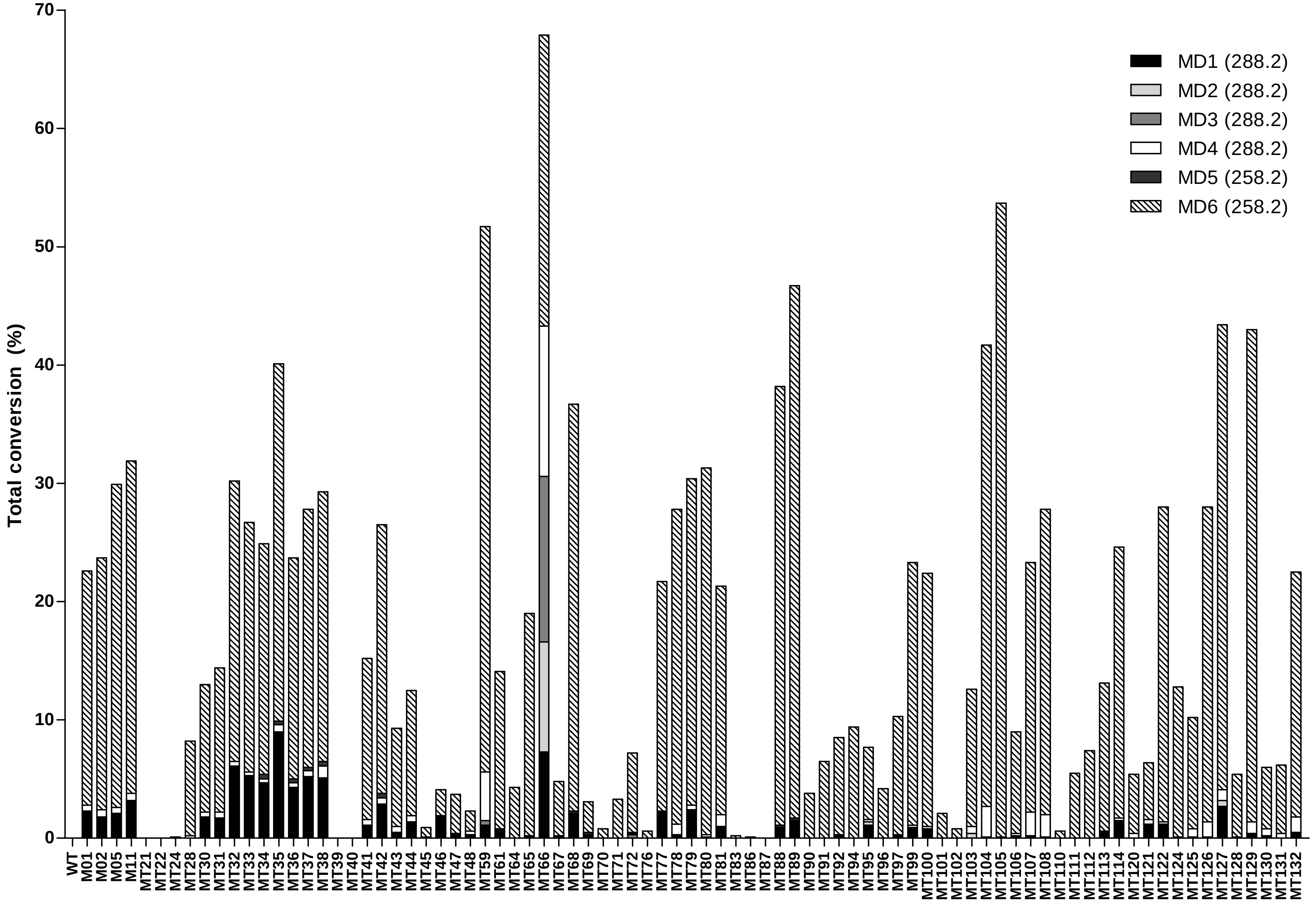

Fig. S3 Product profile and conversion of dextromethorphan by WT enzyme and all CYP BM3 mutants used in this study.

Conversions were determined by integrating MS signals of the parent peak and all metabolites in the corresponding extracted ion chromatograms and are expressed as percentage of the parent metabolized after 60 min. Product selectivity in % was calculated using the MS signals of all metabolites

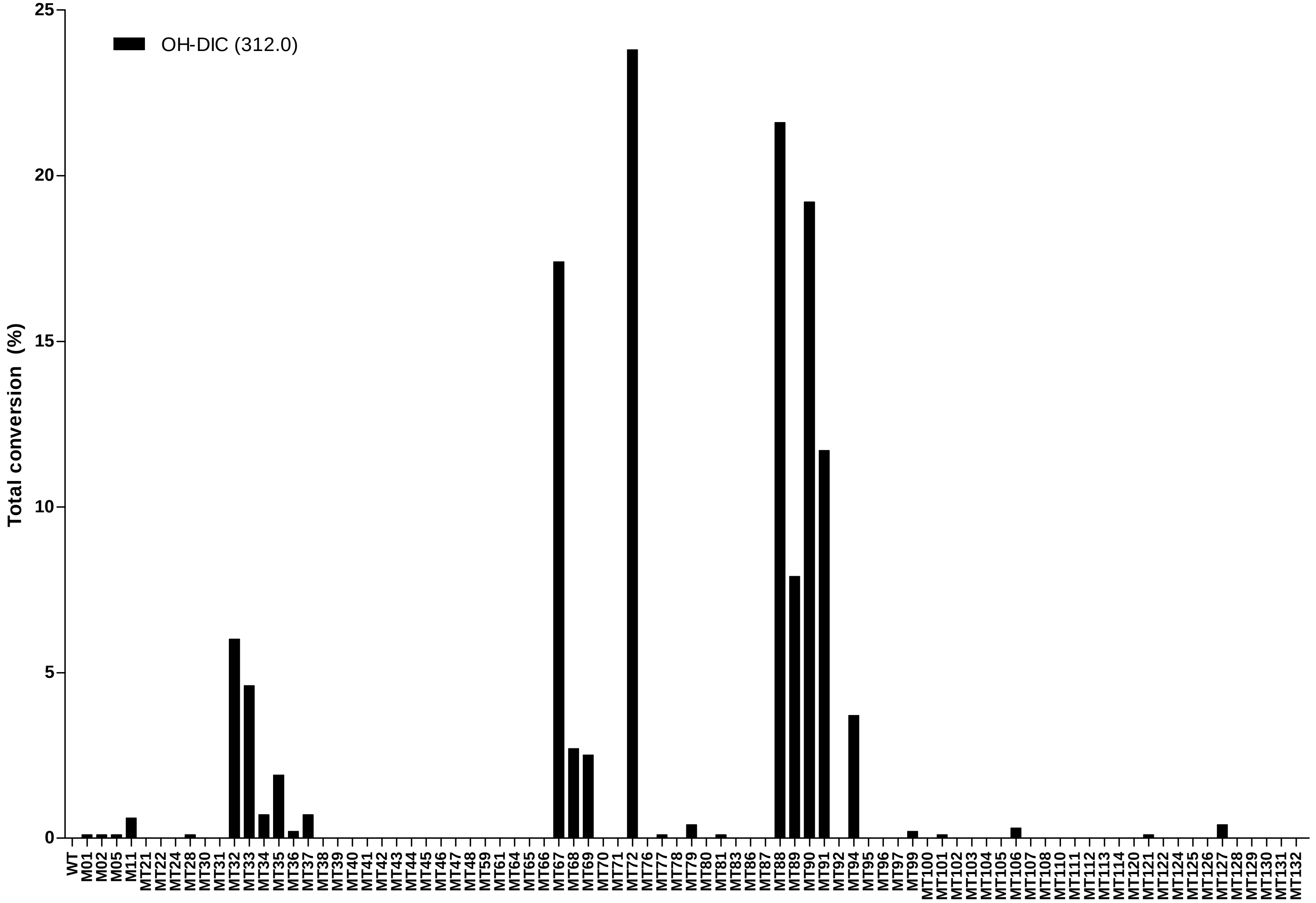

Fig. S4 Product profile and conversion of diclofenac by WT enzyme and all CYP BM3 mutants used in this study. Conversions were

determined by integrating MS signals of the parent peak and all metabolites in the corresponding extracted ion chromatograms and are expressed as percentage of the parent metabolized after 60 min. Product selectivity in % was calculated using the MS signals of all metabolites

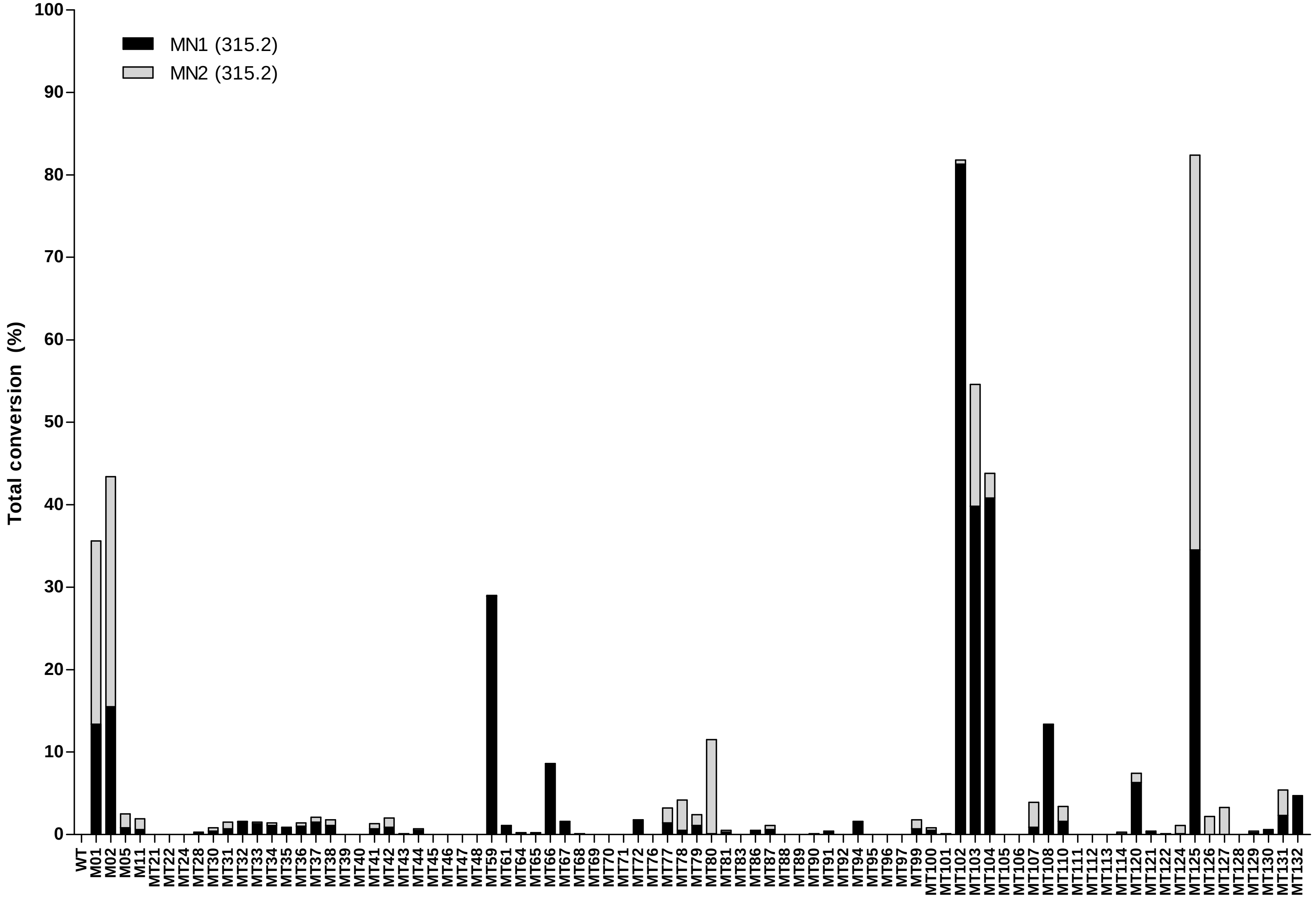

Fig. S5 Product profile and conversion of norethisterone by WT enzyme and all CYP BM3 mutants used in this study. Conversions

were determined by integrating MS signals of the parent peak and all metabolites in the corresponding extracted ion chromatograms and are expressed as percentage of the parent metabolized after 60 min. Product selectivity in % was calculated using the MS signals of all metabolites

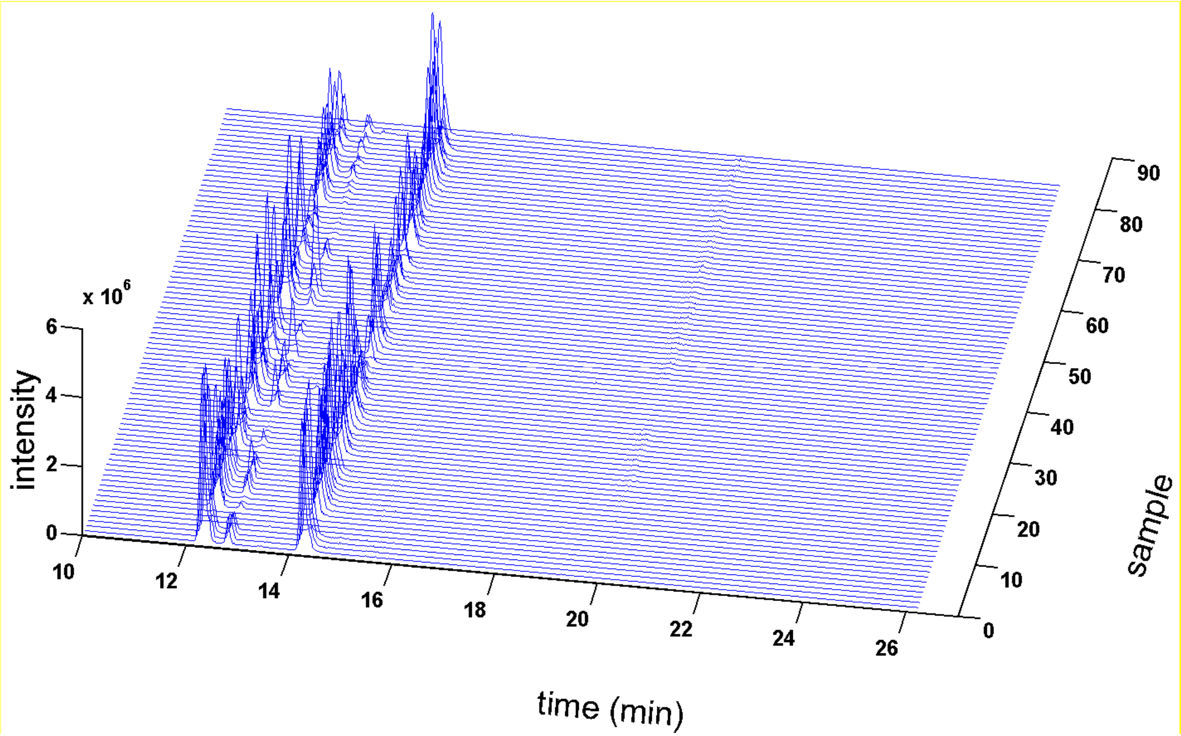

Fig. S6 Mass-trace of *m/z* 294 for all samples. The sample numbers indicate the order of the samples in the data set. In the biplot in Fig. 4A they are linked to the mutant (MT) numbers, used in other figures and tables

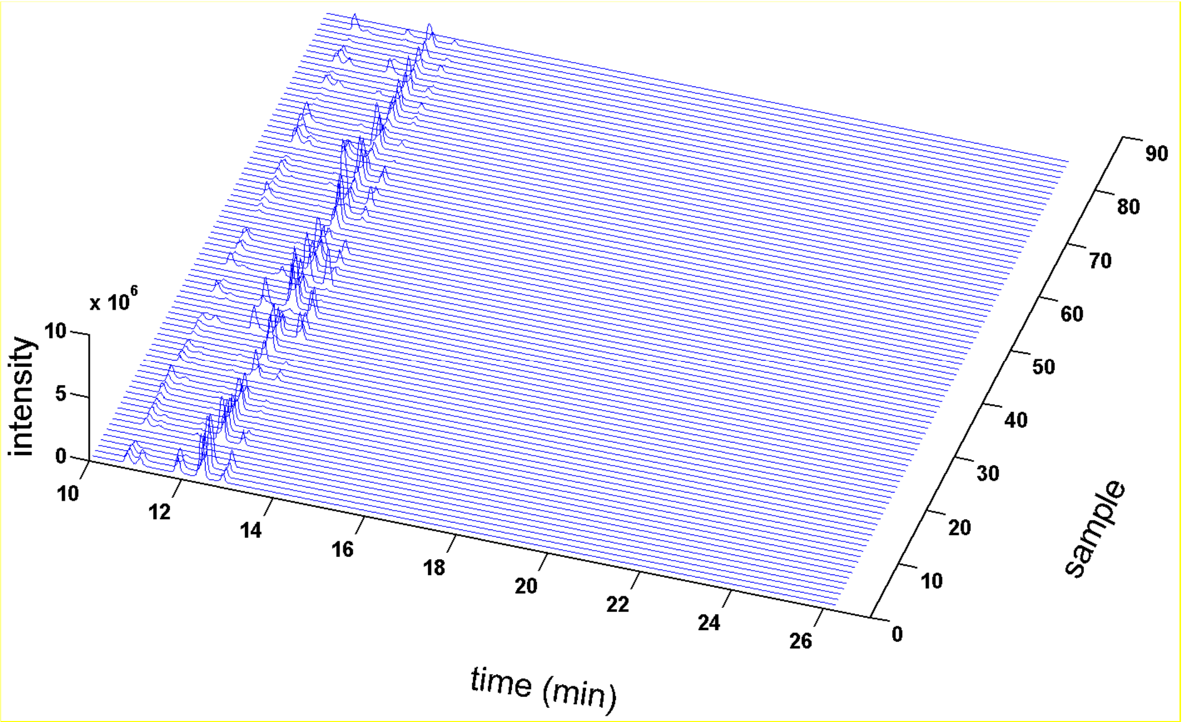

Fig. S7 Mass trace of *m/z* 402 for all samples. The peaks are the BUS metabolites MB1-5. The sample numbers indicate the order of the samples in the data set. In the biplot in Fig 4A they are linked to the mutant (MT) numbers, used in other figures and tables

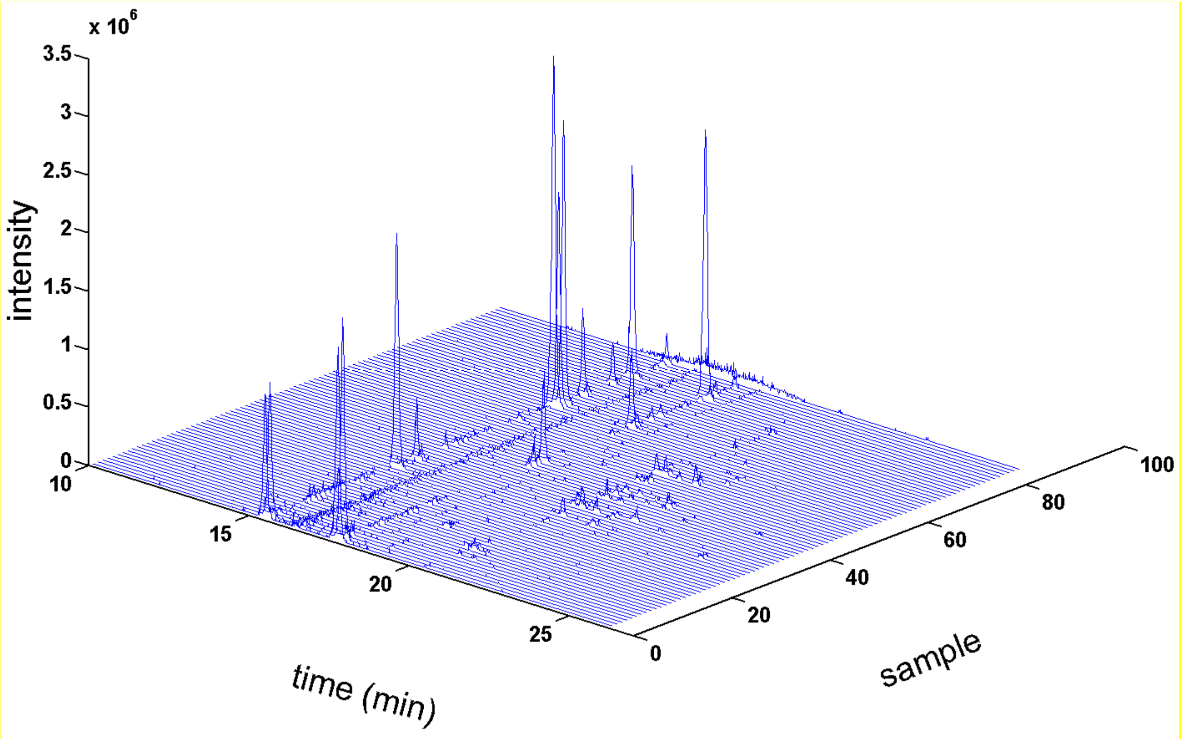

Fig. S8 Mass trace of *m/z* 315 for all samples. The sample numbers indicate the order of the samples in the data set. In the biplot in Fig. 4A they are linked to the mutant (MT) numbers used in other figures and tables

References

1. Rousu T, Herttuainen J, Tolonen A (2010) Comparison of triple quadrupole, hybrid linear ion trap triple quadrupole, time-of-flight and LTQ-Orbitrap mass spectrometers in drug discovery phase metabolite screening and identification in vitro--amitriptyline and verapamil as model compounds. Rapid Commun Mass Spectrom 24:939–57. doi: 10.1002/rcm.4465

2. Reinen J, Van Leeuwen JS, Li Y, et al. (2011) Efficient Screening of Cytochrome P450 BM3 Mutants for Their Metabolic Activity and Diversity toward a Wide Set of Drug-Like Molecules in Chemical Space. Drug Metab Dispos Biol fate Chem 39:1568–1576.

3. Breyer-Pfaff U (2004) The metabolic fate of amitriptyline, nortriptyline and amitriptylinoxide in man. Drug Metab Rev 36:723–46. doi: 10.1081/DMR-200033482

4. Zhu M, Zhao W, Jimenez H, et al. (2005) Cytochrome P450 3A-mediated metabolism of buspirone in human liver microsomes. Drug Metab Dispos 33:500–7. doi: 10.1124/dmd.104.000836

5. Fandiño AS, Nägele E, Perkins PD (2006) Automated software-guided identification of new buspirone metabolites using capillary LC coupled to ion trap and TOF mass spectrometry. J Mass Spectrom 41:248–55. doi: 10.1002/jms.991

6. Rea V, Dragovic S, Boerma JS, et al. (2011) Role of residue 87 in the activity and regioselectivity of clozapine metabolism by drug-metabolizing CYP102A1 M11H: application for structural characterization of clozapine GSH conjugates. Drug Metab Dispos 39:2411–20. doi: 10.1124/dmd.111.041046

7. Strife RJ (1999) Structure elucidation by ion trap sequential mass spectrometry of radical cations formed in low-energy charge-exchange reactions. Rapid Commun Mass Spectrom 13:759–763. doi: 10.1002/(SICI)1097-0231(19990515)13:9<759::AID-RCM555>3.0.CO;2-1

8. Loos WJ, de Graan A-JM, de Bruijn P, et al. (2011) Simultaneous quantification of dextromethorphan and its metabolites dextrorphan, 3-methoxymorphinan and 3-hydroxymorphinan in human plasma by ultra performance liquid chromatography/tandem triple-quadrupole mass spectrometry. J Pharm Biomed Anal 54:387–94. doi: 10.1016/j.jpba.2010.08.033

9. Dragovic S, Boerma JS, Vermeulen NPE, Commandeur JNM (2013) Effect of human glutathione S-transferases on glutathione-dependent inactivation of cytochrome P450-dependent reactive intermediates of diclofenac. Chem Res Toxicol 26:1632–41. doi: 10.1021/tx400204d

10. De Vlieger JS, Kolkman AJ, Ampt KA, et al. (2010) Determination and identification of estrogenic compounds generated with biosynthetic enzymes using hyphenated screening assays, high resolution mass spectrometry and off-line NMR. J Chromatogr B Anal Technol Biomed Life Sci 878:667–674.

11. Rea V, Kolkman AJ, Vottero E, et al. (2012) Active site substitution A82W improves the regioselectivity of steroid hydroxylation by cytochrome P450 BM3 mutants as rationalized by spin relaxation nuclear magnetic resonance studies. Biochemistry 51:750–60. doi: 10.1021/bi201433h

12. Venkatakrishnan K, Moltke LLVON, Greenblatt DJ (2001) Application of the Relative Activity Factor Approach in Scaling from Heterologously Expressed Cytochromes P450 to Human Liver Microsomes : Studies on Amitriptyline as a Model Substrate. 297:326–337.

13. Zhuo X, Gu JUN, Zhang Q, et al. (1999) Biotransformation of Coumarin by Rodent and Human Cytochromes P-450 : Metabolic Basis of Tissue-Selective Toxicity in Olfactory Mucosa of Rats and Mice 1. 288:463–471.

14. Qin C-Z, Ren X, Tan Z-R, et al. (2014) A high-throughput inhibition screening of major human cytochrome P450 enzymes using an in vitro cocktail and liquid chromatography-tandem mass spectrometry. Biomed Chromatogr 28:197–203. doi: 10.1002/bmc.3003

15. Kozakai K, Yamada Y, Oshikata M, et al. (2012) Reliable high-throughput method for inhibition assay of 8 cytochrome P450 isoforms using cocktail of probe substrates and stable isotope-labeled internal standards. Drug Metab Pharmacokinet 27:520–9.

16. Tolonen A, Petsalo A, Turpeinen M, et al. (2007) In vitro interaction cocktail assay for nine major cytochrome P450 enzymes with 13 probe reactions and a single LC/MSMS run: analytical validation and testing with monoclonal anti-CYP antibodies. J Mass Spectrom 42:960–6. doi: 10.1002/jms.1239

17. Jacqz-Aigrain E, Funck-Breantano C, Cresteil T (1993) CYP2D6- and CYP3A-dependent metabolism of dextromethorphan in humans. Pharmacogenetics 3:197–204.

18. Otten JN, Hingorani GP, Hartley DP, et al. (2011) An in vitro, high throughput, seven CYP cocktail inhibition assay for the evaluation of new chemical entities using LC-MS/MS. Drug Metab Lett 5:17–24.

19. Zientek M, Miller H, Smith D, et al. Development of an in vitro drug-drug interaction assay to simultaneously monitor five cytochrome P450 isoforms and performance assessment using drug library compounds. J Pharmacol Toxicol Methods 58:206–14. doi: 10.1016/j.vascn.2008.05.131

20. Dragovic S, Gunness P, Ingelman-Sundberg M, et al. (2013) Characterization of human cytochrome P450s involved in the bioactivation of clozapine. Drug Metab Dispos 41:651–8. doi: 10.1124/dmd.112.050484

21. Tang W, Stearns RA, Wang RW, et al. (1999) Roles of human hepatic cytochrome P450s 2C9 and 3A4 in the metabolic activation of diclofenac. Chem Res Toxicol 12:192–9. doi: 10.1021/tx9802217

22. Korhonen T, Turpeinen M, Tolonen A, et al. (2008) Identification of the human cytochrome P450 enzymes involved in the in vitro biotransformation of lynestrenol and norethindrone. J Steroid Biochem Mol Biol 110:56–66. doi: 10.1016/j.jsbmb.2007.09.025

23. Damsten MC, van Vugt-Lussenburg BM, Zeldenthuis T, et al. (2008) Application of drug metabolising mutants of cytochrome P450 BM3 (CYP102A1) as biocatalysts for the generation of reactive metabolites. Chem Biol Interact 171:96–107.

24. Vottero E, Rea V, Lastdrager J, et al. (2011) Role of residue 87 in substrate selectivity and regioselectivity of drug-metabolizing cytochrome P450 CYP102A1 M11. J Biol Inorg Chem 16:899–912. doi: 10.1007/s00775-011-0789-4

25. Reinen J, Ferman S, Vottero E, et al. (2011) Application of a fluorescence-based continuous-flow bioassay to screen for diversity of cytochrome P450 BM3 mutant libraries. J Biomol Screen 16:239–250.

26. Venkataraman H, Beer SBA De, Geerke DP, et al. (2012) Regio- and Stereoselective Hydroxylation of Optically Active α-Ionone Enantiomers by Engineered Cytochrome P450 BM3 Mutants. Adv Synth Catal 354:2172–2184. doi: 10.1002/adsc.201200067

27. Venkataraman H, Beer SBA De, Bergen LAH Van, et al. (2012) A single active site mutation inverts stereoselectivity of 16-hydroxylation of testosterone catalyzed by engineered cytochrome P450 BM3. Chembiochem 13:520–3. doi: 10.1002/cbic.201100750
